# Assessing Interventions to Manage West Nile Virus Using Multi-Criteria Decision Analysis with Risk Scenarios

**DOI:** 10.1371/journal.pone.0160651

**Published:** 2016-08-05

**Authors:** Valerie Hongoh, Céline Campagna, Mirna Panic, Onil Samuel, Pierre Gosselin, Jean-Philippe Waaub, André Ravel, Karim Samoura, Pascal Michel

**Affiliations:** 1 Groupe de Recherche en Épidémiologie des Zoonoses et Santé Publique, Faculté de médecine vétérinaire, Université de Montréal, Saint-Hyacinthe, Québec, Canada; 2 Institut national de santé publique du Québec, Québec, Canada; 3 Département de médecine sociale et préventive, Université Laval, Québec, Canada; 4 Canadian Field Epidemiology Program, Public Health Agency of Canada, Ottawa, Ontario, Canada; 5 Ouranos, Consortium on regional climatology and adaptation to climate change, Montreal, Quebec, Canada; 6 Group for Research in Decision Analysis, Montréal, Québec, Canada; 7 Département de pathologie et microbiologie, Faculté de médecine vétérinaire, Université de Montréal, Saint-Hyacinthe, Québec, Canada; 8 Université Aube Nouvelle, Ouagadougou, Burkina Faso; 9 National Microbiology Laboratory at Saint-Hyacinthe, Public Health Agency of Canada, Saint-Hyacinthe, Québec, Canada; University of Washington, UNITED STATES

## Abstract

The recent emergence of West Nile virus (WNV) in North America highlights vulnerability to climate sensitive diseases and stresses the importance of preventive efforts to reduce their public health impact. Effective prevention involves reducing environmental risk of exposure and increasing adoption of preventive behaviours, both of which depend on knowledge and acceptance of such measures. When making operational decisions about disease prevention and control, public health must take into account a wide range of operational, environmental, social and economic considerations in addition to intervention effectiveness. The current study aimed to identify, assess and rank possible risk reduction measures taking into account a broad set of criteria and perspectives applicable to the management of WNV in Quebec under increasing transmission risk scenarios, some of which may be related to ongoing warming in higher-latitude regions. A participatory approach was used to collect information on categories of concern to relevant stakeholders with respect to WNV prevention and control. Multi-criteria decision analysis was applied to examine stakeholder perspectives and their effect on strategy rankings under increasing transmission risk scenarios. Twenty-three preventive interventions were retained for evaluation using eighteen criteria identified by stakeholders. Combined evaluations revealed that, at an individual-level, *inspecting window screen integrity*, *wearing light colored*, *long clothing*, *eliminating peridomestic larval sites* and *reducing outdoor activities at peak times* were top interventions under six WNV transmission scenarios. At a regional-level, the use of *larvicides* was a preferred strategy in five out of six scenarios, while use of *adulticides* and *dissemination of sterile male mosquitoes* were found to be among the least favoured interventions in almost all scenarios. Our findings suggest that continued public health efforts aimed at reinforcing individual-level preventive behaviours combined with the application of larvicides to manage the risk of WNV infection are the interventions most acceptable and effective at reaching current management objectives now and under future theoretical transmission risk.

## Introduction

West Nile virus (WNV) is a mosquito-borne flavivirus that first emerged in North America in New York City in 1999 [1,2] and in Canada in 2001 [3,4]. Most WNV infections are asymptomatic, but an important proportion can result in febrile illness with general muscle weakness (approximately 25% of infections) and in rare cases, more severe neurologic symptoms or death (less than 1% of infections) [5]. In the United States of America (US) alone, approximately 42,000 combined cases of neuroinvasive and non-neuroinvasive cases of WNV were reported between 1999 and 2015 with more than 1,700 associated deaths [6]. Over 5,200 cases were reported in Canada between 2002 and 2014, representing a much higher incidence rate relative to reports from the US (given Canada’s approximately 10 times smaller population) [7].

WNV’s emergence in the eastern Canadian province of Quebec in 2002 was linked to climatic conditions that occurred that year [8]. Vector-borne and zoonotic diseases (VBZD), such as WNV, are sensitive to changes in weather and climate [9] and incidence is anticipated to change in response to changes in climate [9–12]. Furthermore, multiple factors including weather are known to affect the transmission and distribution of WNV [13] and climatic projections for Quebec predict rising average temperatures (particularly in winter) and increased average precipitation [14]. As such, early preparedness and planning for current and future VBZD transmission dynamics is a key management strategy for improving public health adaptation to risks posed by climate change.

To date, WNV transmission dynamics have shown themselves to be largely unpredictable in the short term thereby increasing the need to elaborate management strategies that can cover a large range of epidemiologic scenarios [9].Human transmission of WNV in North America follows a seasonal pattern and is the result of a complex ecology of interacting species. The virus is maintained in an enzootic transmission cycle between birds and mosquitoes, primarily of the *Culex* genus, with occasional, dead-end infection in humans and other mammals generally appearing later in the summer season when virus amplification has reached a peak in its avian hosts and mosquito density is at a maximum [5,15–17].

Due to the zoonotic nature and transmission dynamics of WNV, prevention and control opportunities should take place at a number of intervention levels, including: the avian reservoir, the mosquito vector or the human accidental host populations. Known prevention and control strategies range from preventive interventions aimed at individuals, such as the use of mosquito repellents and wearing protective clothing, to vector control interventions, including the application of larvicides or habitat modification measures to reduce mosquito abundance [15–19]. Environmental control interventions aimed at the avian reservoir or the mosquito population have important operational, environmental, and social impacts. These impacts need to be accounted for above and beyond the cost of the interventions alone to ensure feasibility, acceptability and sustainability of the interventions. Although effective WNV vaccines exist for horses; a commercial human WNV vaccine does not yet exist [20–22]. Research is ongoing and a number of promising candidates vaccines that have successfully undergone Phase I and Phase II clinical trials are in development; however, poor perceived cost-benefit of mass vaccination is often cited as the reason for lack of a licensed vaccine for humans at this time [20–22].

Prevention and control of West Nile virus in the province of Quebec (Canada) has primarily consisted of source control of mosquito populations via the use of larvicides, integrated surveillance of humans, animals and mosquitoes, as well as sensitization of the public regarding personal protection measures (21). Uncertainties over the fluctuating yearly numbers of human cases and challenges relative to the perceived high cost of vector control activities in the context of fiscal restraint and government deficits provide ground for periodic re-assessment of the most effective risk reduction strategies. Furthermore, understanding and effectively tackling climate sensitive diseases such as WNV calls for a multidisciplinary perspective and multi-sectoral collaboration [23–25]. Doing so will require transparent approaches that can keep sight of the overarching goal (i.e. reducing public health burden of disease) while taking into account multiple categories of concern, informed by a comprehensive review of available evidence and best-practices [24]. A multi-criteria decision analysis (MCDA) informed approach with multiple stakeholders can help structure reflection and aid in decision-making on the basis of multiple, potentially conflicting criteria (designed to measure specific categories of concern) [26] thereby providing a structured mechanism for multidisciplinary and multi-sectoral collaboration on a decision problem. MCDA enables the ranking of multiple interventions based on a list of stakeholder identified and weighted concerns (i.e. decision criteria) and thus allows for an appreciation of the relative strengths and weakness of various interventions under consideration.

In the current study, preventive interventions for the management of WNV were identified, assessed and ranked using a multi-stakeholder informed MCDA to document effective, favoured and acceptable interventions relating to management of the disease in Quebec under varying increasing transmission risk scenarios in order to help inform future seasonal operational decision making at the provincial level.

## Materials and Methods

**Fig 1:**
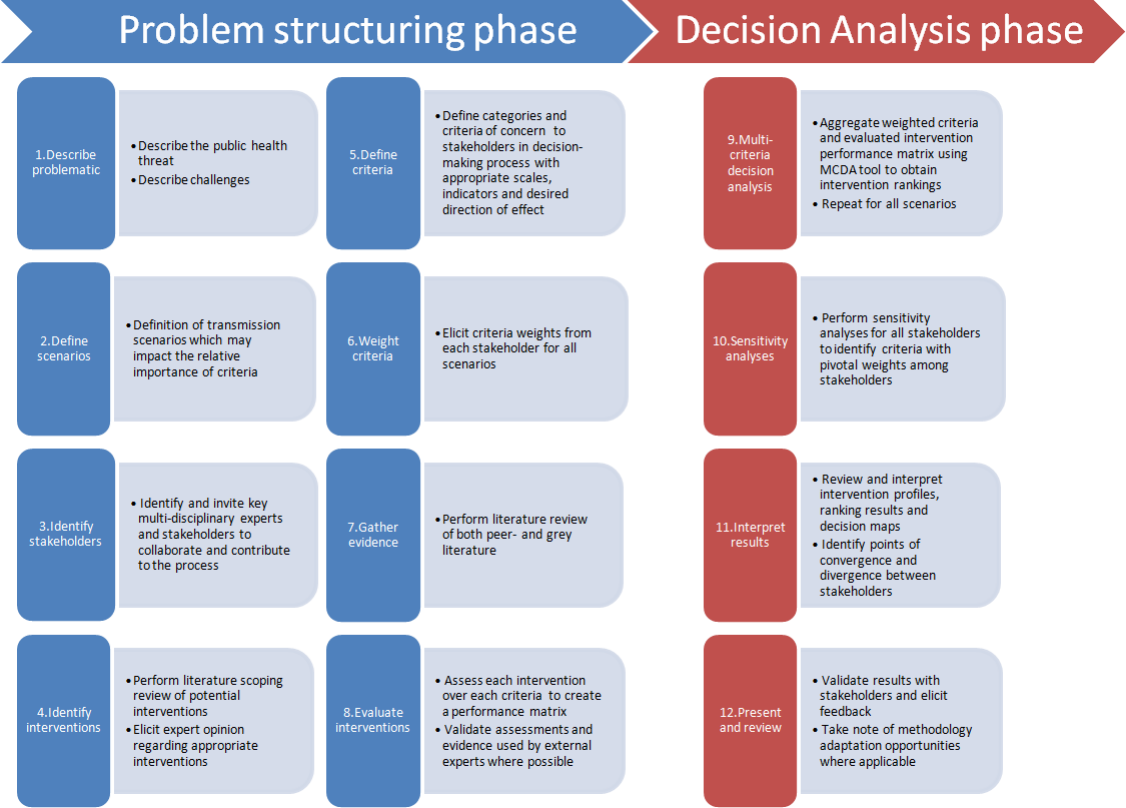
Schematic representation of the MCDA approach (adapted from [27]).

A participatory methodology was adapted from an existing MCDA model for Lyme disease management [27]. MCDA is a formal method that can be used to combine evidence-based information and stakeholder values to support decision-making (Fig 1) [26]. The MCDA method consists first of a ‘problem structuring’ phase. This phase describes the decision problem and identifies a list of management interventions and the important criteria that need to be taken into account when evaluating these interventions. Discussion of the proposed criteria and intervention list by participants ensures exhaustiveness and transparency. Interventions are evaluated using peer-reviewed, grey literature and available data pertaining to all of the retained criteria. Criteria are then weighted by importance by all stakeholders using a standardized form, under different epidemiological transmission scenarios. This allows stakeholders to modify the relative importance of decision criteria (e.g.: incidence reduction vs. cost), depending on the situation they are faced with (ex: low-risk scenario vs. high-risk scenario) and their perspective of the decision problem. The ‘problem structuring’ phase is richest when performed with a varied group of stakeholders, allowing for the integration of multiple concerns (i.e. criteria), and creating the opportunity to build a common understanding of the decision problem. The second stage of the MCDA process is the ‘decision analysis’ phase, where the MCDA analysis tool is used to aggregate the information collected in the first phase (i.e. intervention evaluations and criteria weights) in order to produce a relative ranking of assessed interventions.

### Transmission scenarios

Interventions for the prevention and control of human WNV in the province were evaluated under current and future possible transmission scenarios of WNV in order to support governmental decision making. We constructed six scenarios to reflect potential increases in transmission under current and potential future fluctuations in transmission intensity of the disease. The scenarios themselves are hypothetical and do not reflect historical reality, nor do they reflect a scientific consensus on expected future conditions, rather these scenarios depict fictional, yet climatically plausible WNV transmission scenarios for the province of Quebec [28]. For each scenario, a combination of WNV transmission risk intensity (low, medium and high) and interventions having taken place during the current season were described (Table 1).

**Table 1:** Transmission risk scenarios assessed under the MCDA model for West Nile virus interventions in Quebec.

Scenario	Scenario description	Management context and interventions advocated for WNV season underway
1 **low-risk**—*without interventions* -« Current », end of season, low intensity–Decision for next year	At the end of September, 26 cases declared. All declared cases are symptomatic and distributed among two of the nine sociosanitary regions of Quebec within which human transmission of WNV were previously documented. Clinical presentation of cases was consistent with literature reported symptoms. Passive surveillance of equines (MAPAQ) and of wildlife birds (CQSAS) is coherent with human surveillance data (with respect to the number and geographical distribution of cases). Entomological surveillance data suggests a high density of mosquitoes for the current season, but little WNV found in circulation at present.	Since few WNV cases declared in past two years (< 10) and few resources available to coordinate interventions at beginning of the season, primary intervention strategy for the current season has primarily consisted of providing WNV related information on the ministry website (MSSS)
2 **low-risk**—*with interventions*—« Current », end of season, low intensity—Decision for next year	At the end of September, 26 cases declared. All declared cases are symptomatic and distributed among two of the nine sociosanitary regions of Quebec within which human transmission of WNV were previously documented. Clinical presentation of cases was consistent with literature reported symptoms. Passive surveillance of equines (MAPAQ) and of wildlife birds (CQSAS) is coherent with human surveillance data (with respect to the number and geographical distribution of cases). Entomological surveillance data suggests a high density of mosquitoes for the current season, but little WNV found in circulation at present.	Previous year, 23 cases declared. WNV a concern for Quebec population. Series of interventions carried out at beginning of transmission season. Primary interventions at provincial level: providing WNV related information on ministry website (MSSS). Application of larvicides within risk zones. Calls for vigilance to network medical practitioners. Large scale communication campaign
3 **medium-risk**—*without* interventions—« Outbreak», mid-season, high intensity–Rapid decision for current season	At end of July, 40 symptomatic cases declared to ministry. (Historically, majority of cases occur mid-Aug.-Sep.). 10 cases from regions where no human or animal cases have ever been recorded, suggesting geographical expansion of virus into new zones. Meteorological forecasts predict hot and dry summer. Passive surveillance of equines (MAPAQ) and wildlife (CQSAS) coherent with human surveillance data and suggest acute viral activity compared with data collected over past two years. Among WNV infected horses, 3 declared from regions where no human cases were previously declared and where WNV virus circulation never previously recorded. Entomological surveillance data suggest an increase in mosquito activity and circulation of virus (high density of Culex pipiens and high level of infection). Past two weeks, vector index (number of infected mosquitoes) on rise.	Since few WNV cases declared in past two years (< 10) and few resources available to coordinate interventions at beginning of the season, primary intervention strategy for the current season: providing WNV related information on the ministry website (MSSS)
4 **medium-risk** -*with interventions -* « Outbreak», mid-season, high intensity–Rapid decision for current season	At end of July, 40 symptomatic cases declared to ministry. (Historically, majority of cases occur mid-Aug.-Sep.). 10 cases from regions where no human or animal cases have ever been recorded, suggesting geographical expansion of virus into new zones. Meteorological forecasts predict hot and dry summer. Passive surveillance of equines (MAPAQ) and wildlife (CQSAS) coherent with human surveillance data and suggest acute viral activity compared with data collected over past two years. Among WNV infected horses, 3 declared from regions where no human cases were previously declared and where WNV virus circulation never previously recorded. Entomological surveillance data suggest an increase in mosquito activity and circulation of virus (high density of Culex pipiens and high level of infection). Past two weeks, vector index (number of infected mosquitoes) on rise.	Previous year, 23 cases declared. WNV a concern for Quebec population. Series of interventions carried out at beginning of transmission season. Primary interventions at provincial level: providing WNV related information on ministry website (MSSS). Application of larvicides within risk zones. Calls for vigilance to network medical practitioners. Large scale communication campaign
5 **high-risk**—*without interventions -* « Epidemic», end of season, high intensity—Decision for next year	End of September, 800 symptomatic cases declared. 40 cases from regions where no animal or human cases previously recorded, suggesting a geographical expansion of the virus into new zones. Passive surveillance of equines (MAPAQ) and wildlife (CQSAS) are coherent with human surveillance data and appear to suggest acute viral activity compared with data collected over past two years. Among WNV infected horses, 12 declared from regions where no human cases were previously declared and where virus circulation never previously recorded. Moreover, 72 birds submitted to CQSAS (passive surveillance) tested positive for WNV. Entomological surveillance suggests an increase in mosquito activity and circulation of virus (high density of Culex pipiens and high level of infection). Past four weeks, vector index (number of infected mosquitoes) increasing significantly.	Since few WNV cases declared in last two years (< 10) and few resources available to coordinate interventions at beginning of the season, primary intervention strategy for the current season: providing WNV related information on the ministry website (MSSS)
6 **high-risk**—*with interventions -* « Epidemic», end of season, high intensity—Decision for next year	End of September, 800 symptomatic cases declared. 40 cases from regions where no animal or human cases previously recorded, suggesting a geographical expansion of the virus into new zones. Passive surveillance of equines (MAPAQ) and wildlife (CQSAS) are coherent with human surveillance data and appear to suggest acute viral activity compared with data collected over past two years. Among WNV infected horses, 12 declared from regions where no human cases were previously declared and where virus circulation never previously recorded. Moreover, 72 birds submitted to CQSAS (passive surveillance) tested positive for WNV. Entomological surveillance suggests an increase in mosquito activity and circulation of virus (high density of Culex pipiens and high level of infection). Past four weeks, vector index (number of infected mosquitoes) increasing significantly.	Previous year, 23 cases declared. WNV a concern for Quebec population. Series of interventions carried out at beginning of transmission season. Primary interventions at provincial level: providing WNV related information on ministry website (MSSS). Application of larvicides within risk zones. Calls for vigilance to network medical practitioners. Large scale communication campaign

### Identification of stakeholders

Stakeholders (n = 15) already involved in WNV management from various levels of government, academia as well as from an existing expert committee on WNV in Quebec were invited to participate in the MCDA process in April 2014. Invited stakeholders included individuals from the National institute of public health, Ministry of health and social services, the Public Health Agency of Canada, Ministry of sustainable development, environment and the Fight against climate change, the academic sector, the Quebec center for wildlife health, companies involved in mosquito control operations, Ouranos Consortium for research in climatology and adaptation to climate change and Quebec regional public health authorities. The protocol for this project was reviewed and approved by the Ethical Committee for Health Research of the University of Montreal (Comité d’éthique de la recherche en santé, CERES) (certificate number 14-025-CERES-D). All participants gave informed written email consent for inclusion prior to participation in the study.

### Identification of potential interventions

A comprehensive literature review was conducted to construct a preliminary list of interventions for discussion with stakeholders [5,17,19,29–31]. Interventions including active and passive surveillance, large scale and targeted communication campaigns and various prevention and control interventions were included in this preliminary list. Interventions under development and implementable under both a short and long-term perspective were included in order to provide a range of options to cover all transmission scenarios. A baseline, *status quo* intervention encompassing passive surveillance of human cases and representative of what is currently done to manage WNV in the province was also included (please note that interventions will hereafter be shown in italics in the text while criteria will be shown in “quotes” to ease readability). The proposed interventions were then discussed and validated with participating stakeholders during a focus group discussion. Individual feedback was solicited from all stakeholders following the discussion by means of a Delphi survey during which stakeholders had the opportunity to suggest additional interventions previously missed [32]. Consensus was not explicitly sought during this process; rather stakeholders agreed that an intervention would be retained in the model so long as at least one stakeholder deemed it pertinent to include.

### Identification of decision criteria

Drawing from previous work [27,33], a preliminary list of 15 evaluation criteria, distributed over five categories (“Public Health” criteria, “Social Impact” criteria, “Economic” criteria, “Strategic and Operational”, and “Animal and Environmental Health” criteria) was compiled by the research team. Each criterion was defined with a measurement scale (allowing for a quantitative or qualitative assessment of an intervention), including a direction of desired effect. Linear preference functions were used with all criteria and qualitative assessments were transformed into monotone ascending or descending scales depending on the direction of the desired effect [34]. The relevance of criteria and their measurement scales was discussed and validated with stakeholders. Individual feedback was also solicited via a Delphi survey [32]. Once again, consensus was not explicitly sought regarding retained criteria; rather a criterion was retained so long as at least one stakeholder deemed it pertinent. Weights of zero were permitted by stakeholders to indicate absence of importance for a given criterion during the weighting process (described in the following section).

### Criteria weighting

Stakeholders were asked to weight the relative importance of criteria under all transmission scenarios. Scenarios were presented to stakeholders as hypothetical yet climatically plausible transmission scenarios meant to examine the effect of changing criteria trade-offs under different transmission intensities. For the weighting exercise, stakeholders were given a Microsoft Excel spreadsheet tool and asked to distribute 100 points across the list of criteria included in the model. The more points given to a criterion, the more important this criterion for the stakeholder, thus permitting a relative ranking of criteria. The process was repeated for each of the six scenarios by all stakeholders. Differences in assigned weights were tested between groups of stakeholders using Welch’s *t*-test (unequal variances *t*-test) in R (version 3.3.0) to test for differences in the mean category weights.

### Evaluations of interventions

Assessments were performed for all interventions for all criteria using measurement scales discussed and finalized with stakeholders (see S1 Table). Evaluations were based on existing peer-reviewed evidence, grey literature and available data (see S2 Table for the results of this evaluation and supporting references in S1 Appendix). A comprehensive literature review was performed for all interventions. When data was not available for an evaluation, expert judgment was used. All information relative to the evaluations was compiled into an assessment matrix then revised and discussed by all evaluators. Assessments were further reviewed and validated by external experts with specific field or research experience.

The population specific criterion (“proportion affected”) was assessed as the estimated proportion of the population currently employing these measures for individual-level interventions in population-level analyses. Where data was incomplete, the incidence reduction criterion was assessed as either known to reduce cases or reducing contact between vectors and human hosts. The entomological risk reduction criterion was assessed with regards to having an effect on reducing the population or density of mosquitoes. Data availability and reliability of assessments was tracked to reflect the degree of certainty over provided assessment distinguishing literature based assessment versus expert opinion or field tested result.

### Multi-criteria decision analysis

The evaluations of all interventions were aggregated with criteria weights and analyzed using a multi-criteria analysis tool. The PROMETHEE method (Preference Ranking Organization Method for Enrichment Evaluations) [34] was used to perform multi-criteria analysis with the D-Sight software (version 3.3.2, D-Sight company). Geometrical analysis for interactive aid (GAIA) analysis maps, available with the D-Sight software, were also used to aid in visual interpretation of results [35,36]. Two main sets of analyses were performed, one based on individual-level interventions (n = 11) and the second based on regional-level interventions (n = 10). A subset of mosquito-targeting control measures (n = 8), as well as a subset of the currently available interventions (i.e. interventions ready for deployment within the next year in the province; n = 5), were analysed separately. For the purpose of exploratory comparison, an analysis of combined individual-level and regional-level interventions was also performed. Following this, sensitivity analyses were performed on all criteria and for all stakeholders to examine the robustness of rankings and identify potentially weight-sensitive criteria.

## Results

### Stakeholder consultation and MCDA model construction

Twelve stakeholders (out of 15 invited) consented to participate in the study. Following presentation and discussion of the preliminary lists of interventions and criteria with stakeholders, a final list of 23 interventions (Table 2) and 18 evaluation criteria were retained (Table 3). The identified interventions included individual protective measures, mosquito source reduction measures, adult mosquito control measures, and interventions aimed at the animal reservoir. Four of the twenty-three interventions were not assessed due to insufficient information in the literature to do so (*use of lethal ovitraps*, *reduction in abundance of the main animal reservoir*, *modification of animal reservoir habitat*, and *increased biodiversity at the peridomestic level*). Although communication and surveillance interventions were explicitly recognized as important elements within a VBZD management programme by stakeholders, these interventions were not included in the current model due to concerns regarding the ability to properly assess the efficacy of these interventions under one comprehensive model. The consensus was to explore these interventions separately in a future exercise.

**Table 2:** Potential protection and control interventions for the management of West Nile virus in Quebec.

Scale	Category	Code	Interventions	Description
Individual-level				
	Personal protection measures			
		INT-1	Use of mosquito repellent	Ex.: DEET, citronella, p-menthane-3,8-diol applied to skin
		INT-2	Use of domestic insecticides	Ex.: aerosols, torches, spirales, etc.
		INT-3	Use of alternative technologies	Ex.: automatic insecticide dispensers, electric traps, etc.
		INT-4	Wearing light colored, long clothing	Use of robust and tightly woven fabric
		INT-5	Reducing outdoor activities at peak times	Reduce outdoor activities in high risk areas at dusk and dawn
		INT-6	Reinforcing the immune system	Via healthy living and lifestyle
		INT-7	Inspecting window screen integrity	
		INT-8	Human vaccination	
		INT-9	Wearing insecticide treated clothing*	Insecticide treated clothing
	Source reduction			
		INT-10	Eliminating peridomestic larval sites	Stagnant water, rain water barrels, pails, pool covers, drains
Regional-level				
	Vector targeted source reduction measures			
		INT-11	Modification of natural larval sites	Ex.: water banks, swamps, marshes,
		INT-12	Modification of man-made larval sites	Ex.: treated water basins, reservoirs, damns, roadside ditches, catch basins, underground water canals, vacant and commercial lots, snow disposal sites, used tire sites
		INT-13	Use of parasites and pathogenic micro-organisms	Ex.: nematodes, mushrooms
		INT-14	larvicides	Ground application of larvicides at identified mosquito breeding sites
	Vector targeted population control measures			
		INT-15	Use of mosquito predators	Ex.: birds, bats, fish, insects
		INT-16	Dissemination of sterile males#	Use of sterile male mosquitoes or other compatible insects
		INT-17	Use of lethal ovitraps *†	Traps destined for females with lethal liquid
		INT-18	Use of adulticides	Treatment by truck or plane
	Animal reservoir targeted measures			
		INT-19	Vaccination of animal reservoir *#	Vaccination of the main animal reservoir. Ex.: vaccination of American blackbirds
		INT-20	Reduction of the main animal reservoir *†#	Ex.: controlled reduction of American blackbirds
		INT-21	Modification of animal reservoir habitat *†#	Ex.: move American blackbird dormitories away from inhabited areas
		INT-22	Increase biodiversity at peridomestic level *†#	Ex.: attract other birds near habitat (to reduce circulating levels of the virus)
	Other measures			
		INT-23	Status quo–Human passive surveillance	Encourage research and knowledge transfer regarding control and prevention methods
		INT-24	Large scale communication campaign †	Ex.: media campaign, social media, etc
		INT-25	Targeted communication campaign †	Ex.: health professionals (detection of new cases)
		INT-26	Active surveillance †	Ex.: mosquitoes, birds, human cases

* Interventions added following discussion with stakeholders

† Interventions not assessed due to insufficient data or following discussion with stakeholders

# Interventions in development, not currently implementable

Note: *Interventions* are listed in *italics* when referenced in the text to distinguish from “criteria” which are listed in “quotes”

**Table 3:** Criteria for the management of West Nile virus in Quebec.

Category	WNV criteria	Description
Public Health Criteria (PHC)		
	PHC1—Incidence reduction	Reduction in incidence of human cases
	PHC2—Entomological risk reduction	Reduction of entomological risk
	PHC3 –Physical health impact	Impacts to human physical health
	PHC4—Mental health impact	Impacts to human mental health
	PHC5 –Social equity*	Impact on social equity
	PHC6 –Reduction of circulating virus	Reduction in level of circulating virus in animal reservoir
	PHC7 –Proportion affected	Proportion of population that benefits from the action
Social Impact Criteria (SIC)		
	SIC1 –Public acceptance	Level of public acceptance
	SIC2 –Impact to credibility	Impact to confidence in and credibility of organisation in charge
Economic Criteria (ECC)		
	ECC1 –Government cost	Cost to the government
	ECC2 –Municipal cost	Cost to municipalities
	ECC3 –Individual cost	Cost to individuals and private sector
Strategic & Operational Criteria (SOC)		
	SOC1—Delay	Delay before appearance of desired effect
	SOC2 –Complexity	Institutional and operational complexity of the action
	SOC3 –Sustainability *	Sustainability of the action
	SOC4 –Other policy impact*	Impact on other public policies
Animal & Environmental Criteria (AEC)		
	AEC1 –Animal health impact	Impact on animal health
	AEC2 –Environmental impact	

* Criteria added following discussion with stakeholders

Note: Criteria are listed in “quotes” when referenced in the text to distinguish from *interventions* which are listed in *italics*

### Criteria weighting

Stakeholder weights for the criteria under all scenarios are included in the supporting information (see S3–S5 Tables for the individual weighting results). The criteria deemed most important (most points attributed per criterion by stakeholders), were predominantly criteria related to the “Public Health” category, followed by the “Economic” category or the “Strategic and Operational” criteria category. In nearly all transmission scenarios, “Animal and Environmental Health” criteria ranked lowest, with fewest weights attributed by stakeholders. Within the “Public Health” category, a majority of weights were attributed to the “incidence reduction” criterion, and “physical health impact” criterion. Within the “Social Impact” category, the *“*credibility impact” criterion received the highest weight in most scenarios. Within the “Economic” criteria category, the “government cost” criterion received the highest weight. Within the “Strategic and Operational” criteria category, the “delay” criterion was given highest weight for medium and high scenarios. Finally, in the “Animal and Environmental Health” criteria category, the “environmental impact” criterion was given the highest weight for all scenarios.

### Global results

**Fig 2:**
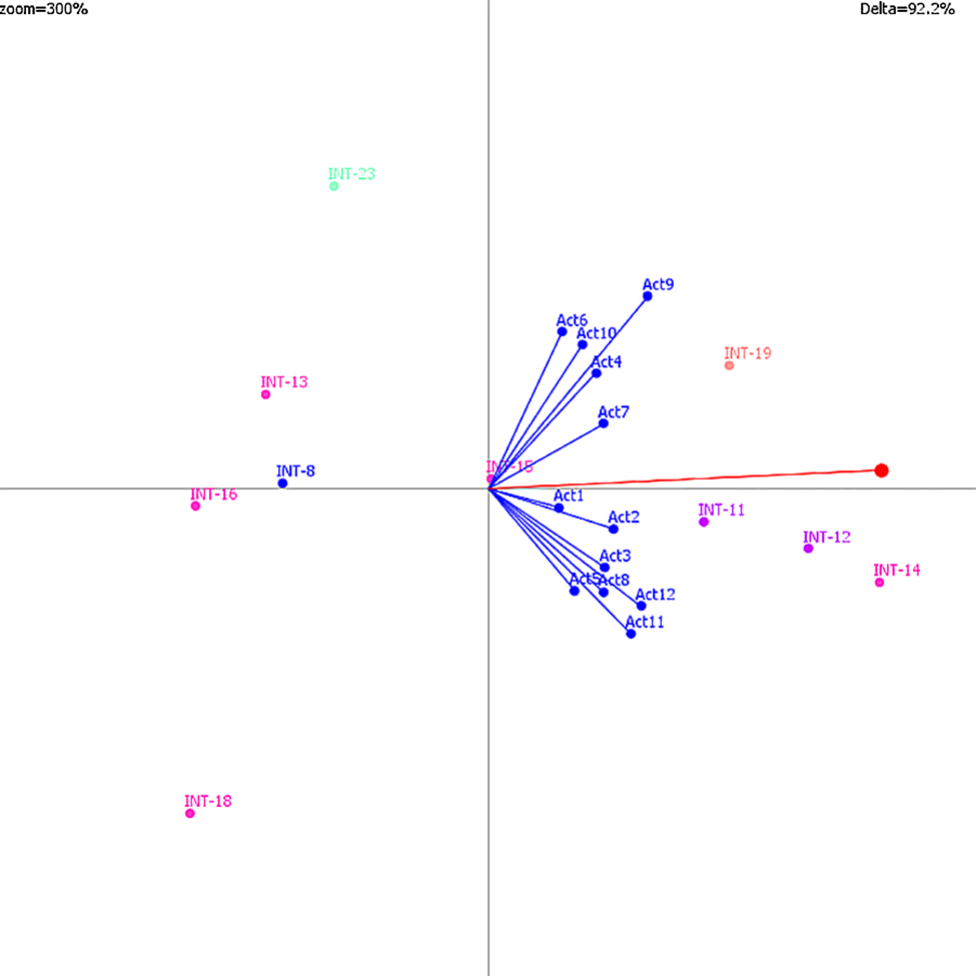
GAIA decision map for regional-level model under scenario 6 (high-risk transmission with interventions). Vector points Act1 through Act12 represent the 12 stakeholders in the model. Points INT-8-23 represent the various Interventions under consideration in this analysis (see Table 2). The red vector indicates the group decision axis with preferred direction indicated by the red dot. Proximity of intervention points along the decision axis represents group ranking preference for these interventions. The relatively proximity of all stakeholder points in the same general direction as the decision axis indicates that all stakeholders are generally in agreement with the group decision axis, and no stakeholder is diametrically opposed to this decision. The close proximity of all stakeholders to one another furthermore indicates fairly strong consensus between stakeholders. There are two slightly divergent coalitions of stakeholders (1^st^ group consists of stakeholders above the decision axis and the 2^nd^ group consists of those below) indicating that these two groups have slightly different perspectives with regards to their criteria weighting, but these differences in perspective are not in conflict with the group decision axis. (Zoom = 300% and Delta = 92.2%, indicates that 92.2% of the information is conserved in the two-dimensional representation of this decision map).

A strong level of congruence was generally observed among weights expressed by stakeholders across all scenarios. The high-risk transmission scenario analysis of regional-level interventions illustrates this (Fig 2). In Fig 2, two semi-coalitions of stakeholders can be observed consisting in one case of stakeholders 4,5,7,9 and 10 and in the second case of stakeholders 1,2,3,5,8,11 and 12. Stakeholder positions are generally all pointing in the same direction as the decision axis indicating that no stakeholders is in direct opposition to the group consensus; however slight differences between these two groups of stakeholder weights can be observed. A statistical comparison of weights (Welch’s *t*-test, unequal variances) revealed that these two groups of stakeholders had significant differences in weights for the Social impact category (p = 0.015) as well as the Animal and Environmental Health criteria category (p = 0.04). From an organizational standpoint, stakeholders in the 2nd coalition consist of a mix of organizations including public health, wildlife and environmental management. The 1st coalition consists of a mix of wildlife and public health related organizations. The bigger difference between these two groups may be their spatial planning mandates with stakeholders in coalition 2 having more involvement in daily field operations and stakeholders from coalition 1 being more involved at a regional planning scale, though not strictly so. Both points of view are important to take into account and despite their differences in weighting; there is a consensus with regards to recommended interventions. Stakeholder positions were seen to converge under scenarios of increasing severity. Sensitivity analyses were performed to examine the robustness of weights given by stakeholders to criteria in the models and their effect on the overall rankings. The criteria most sensitive to stakeholder weights primarily consisted of criteria from the “Public Health” category, as well as the “credibility impact” criterion, “individual cost” criterion and “government cost” criterion.

### Ranking of individual-level interventions

**Fig 3:**
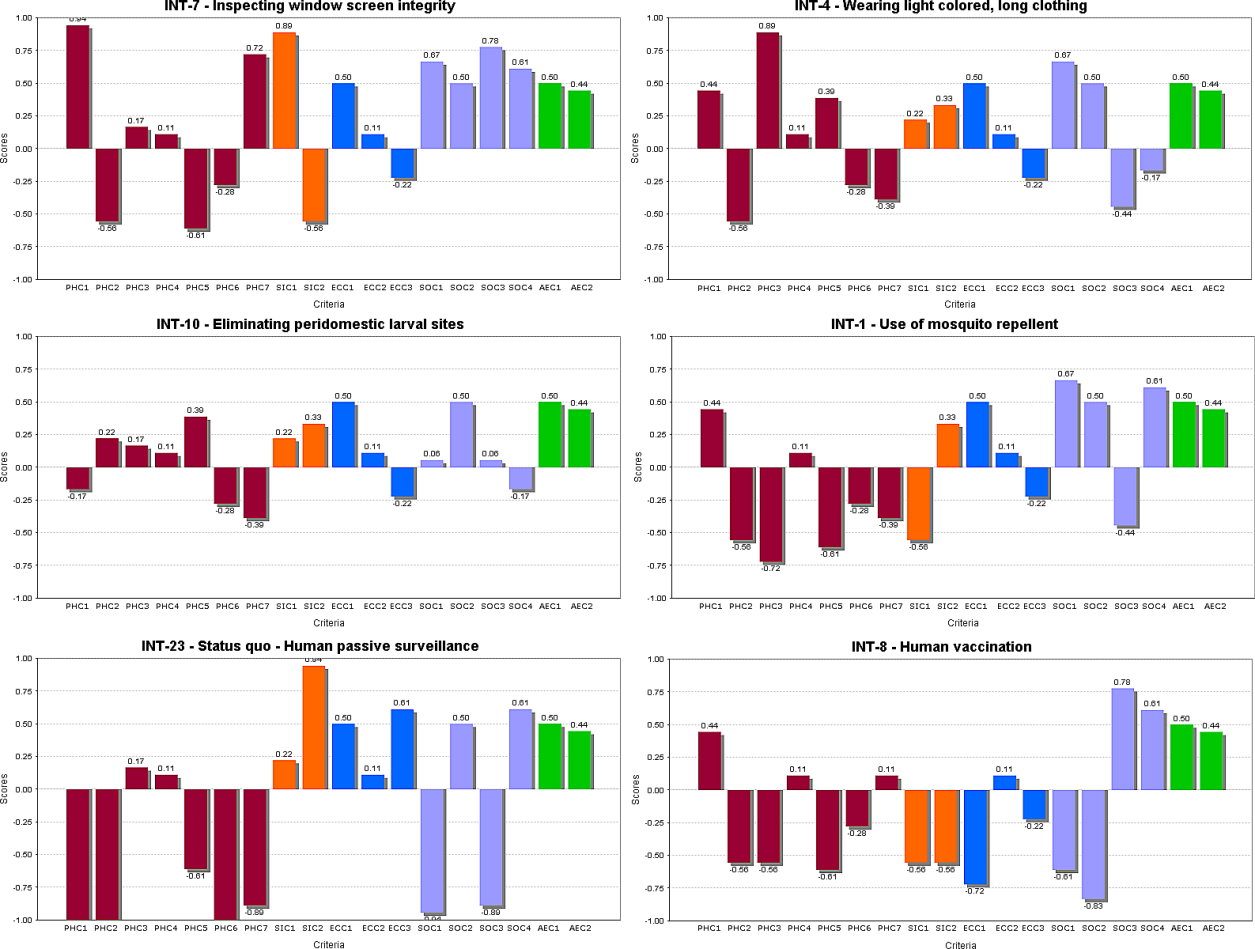
Intervention profiles for six individual-level protection interventions. Each bar represents one of the criteria included in the model. Values along the vertical axis indicate the scores received for the intervention on a particular criterion. Values above zero indicate good performance of the intervention for that criterion based on evaluation scores and conversely, values below zero indicate “poor” relative performance. Criteria bar color codes: red: Public Health criteria; orange: Social Impact criteria; blue: Economic criteria; purple: Strategic and Operational criteria; green: Animal and Environmental Health criteria. (Please refer to supplementary material for all other intervention profiles).

The top four ranked personal protection interventions, *inspecting window screen integrity*, *wearing lightly colored clothing*, and *eliminating peridomestic mosquito larval sites*, *reducing outdoor activities at peak times*, were identical across all scenarios (Table 4). These rankings are based on evidence-based assessment scores combined with stakeholder assigned weights. Fig 3 shows how *Inspecting window screen integrity* scores high on a majority of criteria with the exception of “entomological risk reduction”, “reduction of circulating virus” and “social equality” where it received lower scores. The second and third ranked interventions, *wearing light colored clothing* and *eliminating peridomestic larval sites*, also scored highly on a majority of criteria (Fig 3).

**Table 4:** Ranking of the individual-level protection interventions.

Scenarios	Low risk				Medium risk				High risk			
	1		2		3		4		5		6	
Intervention	Rank	Net Flow	Rank	Net Flow	Rank	Net Flow	Rank	Net Flow	Rank	Net Flow	Rank	Net Flow
INT-01 Use of mosquito repellent	5	-0	5	-0	6	-0	6	-0	6	-0.02	6	-0
INT-02 Use of domestic insecticides	8	-0.1	8	-0.1	7	-0.1	7	-0.1	8	-0.11	8	-0.1
INT-03 Use of alternative technologies	9	-0.1	9	-0.1	8	-0.1	9	-0.1	9	-0.11	9	-0.1
INT-04 Wearing light colored, long clothing	2	0.19	2	0.17	2	0.22	2	0.22	2	0.23	2	0.22
INT-05 Reduction of activities at peak times	4	0.09	4	0.07	4	0.09	4	0.08	4	0.09	4	0.08
INT-06 Reinforcing the immune system	6	-0	7	-0.1	9	-0.1	8	-0.1	7	-0.05	7	-0.1
INT-07 Inspecting window screen integrity	1	0.22	1	0.23	1	0.25	1	0.25	1	0.27	1	0.25
INT-08 Human vaccination	11	-0.2	11	-0.2	11	-0.2	11	-0.2	10	-0.19	10	-0.2
INT-09 Wearing insecticide treated clothing	7	-0	6	-0	5	0.01	5	0	5	0.03	5	0.01
INT-10 Eliminating peridomestic larval sites	3	0.12	3	0.11	3	0.11	3	0.1	3	0.10	3	0.11
INT-23 Status quo	10	-0.2	10	-0.1	10	-0.2	10	-0.2	11	-0.23	11	-0.2

The least favoured interventions among this subset varied slightly from one transmission scenario to another, but generally included: *use of alternative technologies*, *human vaccination* and *status quo* (Table 2). Examination of the profiles for the bottom ranked interventions, *status quo* and *human vaccination*, (Fig 2) shows how these interventions score poorly on most criteria including many “Public Health” criteria, a category consistently weighted highly by all stakeholders. *Human vaccination* in particular scores poorly over many criteria, notably “entomological risk reduction”, “physical health impact”, “social equity” (if not covered by universal health care, then some costs must be incurred by the general public for vaccination), “public acceptance”, “credibility impact”, “government cost”, “individual cost”, “delay”, and “complexity” (highly complex since licensed human vaccine not yet available).

### Ranking of regional-level interventions

In the model containing regional-level management interventions (Table 5), the top three identified interventions were consistently: *larvicides*, v*accination of animal reservoir* and *modification of man-made larval sites* with small variations in the order of these interventions depending on the scenarios. Examination of regional-level intervention profiles showed *larvicides*, *vaccination of animal reservoir* and *modification of man-made larval sites*, to be top scorers over most of the criteria, although *Larvicides* scored less well on the “government cost”, “complexity”, “other policy impact”, “animal health impact” and “environmental impact” criteria (see S2 and S3 Figs). The *vaccination of animal reservoir* intervention was found to score less well on the “incidence reduction” criterion compared to *larvicides*, but scored relatively well on other criteria “reduction of circulating virus” criterion in particular. The *modification of man-made larval sites* intervention scored less well on “Economic” criteria, “Strategic and Operational” criteria and the “Animal and Environmental Health” criteria.

**Table 5:** Ranking of the regional-level management interventions.

Scenarios	Low risk				Medium risk				High risk			
	1		2		3		4		5		6	
Intervention	Rank	Net Flow	Rank	Net Flow	Rank	Net Flow	Rank	Net Flow	Rank	Net Flow	Rank	Net Flow
INT-08 Human vaccination	10	-0.18	8	-0.13	8	-0.17	9	-0.21	7	-0.13	7	-0.15
INT-11 Modification of natural larval sites	5	0.01	6	-0.01	4	0.12	4	0.17	4	0.13	4	0.13
INT-12 Modification of man-made larval sites	3	0.10	3	0.08	3	0.18	3	0.18	2	0.22	2	0.21
INT-13 Use of parasites and pathogenic micro-organisms	7	-0.07	7	-0.07	7	-0.13	7	-0.13	8	-0.16	8	-0.15
INT-14 larvicides	1	0.21	2	0.19	1	0.25	1	0.28	1	0.29	1	0.27
INT-15 Use of mosquito predators	4	0.09	4	0.05	5	0	5	0.02	5	0.02	5	0.01
INT-16 Dissemination of sterile males	8	-0.15	9	-0.16	9	-0.19	10	-0.21	10	-0.21	9	-0.21
INT-17 Use of adulticides	9	-0.17	10	-0.22	10	-0.21	8	-0.19	9	-0.19	10	-0.21
INT-18 Vaccination of animal reservoir	2	0.20	1	0.24	2	0.22	2	0.19	3	0.15	3	0.19
INT-23 Status quo–Human passive surveillance	6	-0.05	5	0.02	6	-0.07	6	-0.10	6	-0.12	6	-0.1

The ordering of the bottom three interventions included: *use of adulticides*, *dissemination of sterile males*, and *human vaccination* in the low and medium-risk scenarios. For the high-risk scenarios, the bottom ranked interventions changed to include *use of parasites and pathogenic microorganisms* instead of *human vaccination*.

### Ranking of mosquito-targeting and currently available interventions

Among the mosquito-targeting interventions (Table 6), the top two ranked were *larvicides* and *modification of man-made larval sites*. This was followed by *use of mosquito predators* in the first two scenarios and *modification of natural larval sites* across remaining scenarios. The bottom three ranked interventions included, in order, the *use of parasites and pathogenic microorganisms*, the *dissemination of sterile males* and the *use of adulticides* for the low- and medium- risk scenarios with the ordering of the last two interventions reversed for the two high-risk scenarios.

**Table 6:** Ranking of the mosquito-targeted control measures.

Scenarios	Low risk				Medium risk				High risk			
	1		2		3		4		5		6	
Intervention	Rank	Net Flow	Rank	Net Flow	Rank	Net Flow	Rank	Net Flow	Rank	Net Flow	Rank	Net Flow
INT-11 Modification of natural larval sites	4	0.01	5	0.00	3	0.12	3	0.17	3	0.12	3	0.13
INT-12 Modification of man-made larval sites	2	0.12	2	0.10	2	0.20	2	0.18	2	0.23	2	0.23
INT-13 Use of parasites and pathogenic micro-organisms	6	-0.08	6	-0.05	6	-0.13	6	-0.14	6	-0.16	6	-0.15
INT-14 Larvicides	1	0.23	1	0.21	1	0.27	1	0.29	1	0.30	1	0.29
INT-15 Use of mosquito predators	3	0.09	3	0.06	4	0.00	4	0.01	4	0.02	4	0.01
INT-16 Dissemination of sterile males	7	-0.16	7	-0.15	7	-0.19	8	-0.22	8	-0.22	8	-0.21
INT-17 Use of adulticides	8	-0.17	8	-0.21	8	-0.20	7	-0.18	7	-0.18	7	-0.20
INT-23 Status quo–Human passive surveillance	5	-0.04	4	0.04	5	-0.06	5	-0.11	5	-0.11	5	-0.09

In the analysis of currently available to deploy regional-level management interventions (Table 7), the ranking did not change for any of the six scenarios and included *larvicides*, *modification of man-made larval sites*, *status quo*, *use of parasites and pathogenic microorganisms* and *use of adulticides* in the listed order.

**Table 7:** Ranking of the currently available management interventions.

Scenarios	Low risk				Medium risk				High risk			
	1		2		3		4		5		6	
Intervention	Rank	Net Flow	Rank	Net Flow	Rank	Net Flow	Rank	Net Flow	Rank	Net Flow	Rank	Net Flow
INT-12 Modification of man-made larval sites	2	0.07	2	0.05	2	0.14	2	0.12	2	0.17	2	0.17
INT-13 Use of parasites and pathogenic micro-organisms	4	-0.06	4	-0.05	4	-0.11	4	-0.11	4	-0.14	4	-0.13
INT-14 Larvicides	1	0.23	1	0.21	1	0.27	1	0.31	1	0.29	1	0.29
INT- Use of adulticides	5	-0.21	5	-0.26	5	-0.25	5	-0.22	5	-0.24	5	-0.25
INT-23 Status quo–Human passive surveillance	3	-0.03	3	0.04	3	-0.05	3	-0.09	3	-0.09	3	-0.07

### Ranking of combined individual- and regional-level interventions

In the combined model of individual- and regional-level interventions (Table 8), *inspecting window screens* and *wearing lightly colored clothing* were always ranked 1^st^ and 2^nd^. This was most often followed by *larvicides* in all but the low-risk scenario 2 where it was replaced by *eliminating peridomestic larval sites*. The bottom three ranked interventions most often included *use of parasites and pathogenic microorganisms*, *dissemination of sterile males*, and *adulticides*.

**Table 8:** Ranking of the individual-level protection and regional-level management interventions combined.

Scenarios	Low risk				Medium risk				High risk			
	1		2		3		4		5		6	
Intervention	Rank	Net Flow	Rank	Net Flow	Rank	Net Flow	Rank	Net Flow	Rank	Net Flow	Rank	Net Flow
INT-01 Use of mosquito repellent	9	0.00	7	0.06	9	0.05	10	0.02	10	0.02	10	0.02
INT-02 Use of domestic insecticides	11	-0.04	9	0.02	11	0.01	11	-0.02	12	-0.06	11	-0.04
INT-03 Use of alternative technologies	14	-0.11	12	-0.04	12	-0.05	12	-0.07	14	-0.12	14	-0.09
INT-04 Wearing light colored, long clothing	2	0.19	2	0.22	2	0.27	2	0.25	2	0.27	2	0.25
INT-05 Reduction of activities at peak times	5	0.11	4	0.13	4	0.14	5	0.12	4	0.13	5	0.11
INT-06 Reinforcing the immune system	13	-0.06	10	-0.02	13	-0.07	14	-0.08	11	-0.06	12	-0.06
INT-07 Inspecting window screen integrity	1	0.32	1	0.34	1	0.34	1	0.33	1	0.35	1	0.33
INT-08 Human vaccination	17	-0.15	16	-0.15	16	-0.19	17	-0.21	15	-0.14	15	-0.16
INT-09 Wearing insecticide treated clothing	10	-0.02	8	0.03	8	0.05	9	0.07	8	0.05	9	0.03
INT-10 Eliminating peridomestic larval sites	6	0.11	3	0.14	5	0.14	4	0.12	6	0.11	4	0.12
INT-20 Modification of natural larval sites	12	-0.06	15	-0.10	10	0.02	8	0.07	9	0.05	8	0.05
INT-21 Modification of man-made larval sites	7	0.01	11	-0.04	7	0.05	7	0.07	5	0.12	6	0.11
INT-22 Use of parasites and pathogenic micro-organisms	15	-0.12	17	-0.16	17	-0.21	16	-0.19	17	-0.22	17	-0.21
INT-23 Larvicides	3	0.16	6	0.10	3	0.14	3	0.18	3	0.20	3	0.18
INT-24 Use of mosquito predators	8	0.01	13	-0.07	14	-0.11	13	-0.08	13	-0.07	13	-0.07
INT-25 Dissemination of sterile males	18	-0.16	18	-0.22	18	-0.25	18	-0.24	19	-0.26	18	-0.25
INT-27 Use of adulticides	19	-0.19	19	-0.26	19	-0.28	19	-0.25	18	-0.24	19	-0.25
INT-28 Vaccination of animal reservoir	4	0.13	5	0.11	6	0.09	6	0.08	7	0.06	7	0.09
INT-32 Status quo—human passive surveillance	16	-0.12	14	-0.08	15	-0.15	15	-0.17	16	-0.19	16	-0.17

## Discussion

This study has demonstrated adaptation planning for management of WNV under various increasing transmission risk scenarios using multi-criteria decision analysis (MCDA). To the best of our knowledge, this is the first study to use MCDA for management planning of WNV. Aenishaenslin and colleagues (2013) had previously demonstrated the possibility of MCDA use for management of Lyme disease emergence in Canada and had suggested that general criteria categories exist that are suitable for VBZD management at large [27]. The categories retained in our study are consistent with previous multi-stakeholders concerted decisions that have taken place in public health over the past 20 years [33,37,38]. Our study further supports the application of MCDA for VBZD and reinforces the notion of common categories of concern to consider in VBZD management. Additionally, our study has shown how many of these concerns remain relevant under various increasing transmission risk scenarios, many of which are coherent with a change in transmission intensity that could occur with climate change. Indeed, substantial warming in higher-latitude regions will likely open up new terrain for some infectious diseases that are limited at present by low temperature boundaries [39].

The degree of concern (weights) attributed to different criteria by stakeholders was shown to vary with transmission intensity of scenarios. This was expected as we anticipated that an increasing number of reported cases in the scenarios would lead to increased concern for public health and social impact related considerations thereby triggering a trade-off among remaining criteria. A similar result was found in the Lyme disease study [27]. A priori hypotheses around economic cost trade-offs were that as WNV incidence increased, costs would become less of a concern with regards to investment in interventions. Indeed, this pattern is observed but is more apparent when scenarios 1,3,and 5 (scenarios without interventions performed during the current season) are compared together versus scenarios 2,4 and 6 (scenarios where interventions have been carried out during the current season). Despite the decreasing importance of cost under increased transmission intensity, important differences in intervention rankings were not observed. The ranking of interventions was found to vary under different scenarios and among the different models. This was also expected since changes in weights affect rankings. Intervention profiles can be examined to further understand the relative rankings of interventions independently of stakeholder assigned weights (see supplementary material for comprehensive coverage of profiles). Model rankings and interpretation are discussed in more detail in the following sections.

### Individual-level protection model

The relative rankings of individual-level interventions were generally not found to vary considerably across the scenarios (low to higher risk transmission). This stability suggests specific protective behaviors that remain effective and acceptable and should continue to be promoted in communication campaigns in order to reinforce adaptive capacity under increasing transmission intensity.

The individual-level model results observed where *inspection of window screens*, *wearing light colored clothing*, *eliminating peridomestic larval sites* and *reducing outdoor activities at peak times* were highly ranked and *use of alternate technologies*, *human vaccination* and *status quo* were lower ranked are consistent with primary prevention messages already included in Quebec WNV communication campaigns as well as other Canadian and the US ones [40–42]. These messages are also consistent with personal protection methods prescribed within integrated vector management programs in Europe [18]. The *inspection of window screens* in particular was the most highly ranked intervention at this level and indeed is already a common and well accepted practice in most homes in the province of Quebec [43]. As such few if any financial costs are expected to be associated with the promotion of this strategy; however, individuals without sufficient economic means may be less likely to replace or purchase window screens. Examination of the relative strengths and weaknesses of interventions via their intervention profiles (see Fig 3 and S1 Fig) illustrates how a comprehensive public health strategy can be built that addresses all concerns raised by stakeholders. For example, the second and third ranked interventions, *wearing light colored clothing* and *eliminating peridomestic larval sites*, which also ranked highly, are complementary to the *inspecting window screen integrity* intervention as they score well on criteria where *inspecting window screens* performed less well (Fig 3).

### Regional-level management model

Overall, the rankings of regional-level interventions were found to vary more than individual-level interventions across the increasing transmission intensity scenarios. The positional stability of top ranked interventions here too suggests specific actions to manage WNV effectively that remain acceptable across a range of transmission dynamics. The positional change of other interventions such as *vaccination* or *modification of natural mosquito larval sites*, under the higher transmission risk scenarios suggests increased acceptability of potentially more controversial interventions under these conditions. Periodic re-evaluations are warranted as additional information becomes available for these interventions.

Evaluated regional-level interventions were primarily vector targeted with the exception of the *vaccination* (human and animal) and *status quo* (human passive surveillance) interventions. Top ranking interventions included *larvicides*, *vaccination of animal reservoir* and *modification of man-made larval sites* having scored highly on most criteria but with important trade-offs on other criteria. For example, *Larvicides* scored poorly on cost, operational complexity and environmental criteria. Mosquito control programs are costly and complex to operate as they require entomological surveillance programs, well-trained staff and infrastructure [17] and repeated application in order to maintain effectiveness [44,45]. Nevertheless, vector control remains key to effective vector borne disease management [46]. While the *vaccination of animal reservoir* intervention was highly ranked, the inclusion of a criterion explicitly targeting the level of circulating virus in the animal reservoir may explain the high ranking of this strategy as it is the only measure that directly acts on this aspect of transmission. A few studies have demonstrated success with this measure [47–49] but for the time being, it remains a hypothetical intervention for the province of Quebec. With regards to *man-made larval sites*, *s*tudies have found that proximity to certain types of structures such as combined sewer overflow systems have been significantly associated with high rates of WNV infection in humans and corvids; however, construction and modification of major infrastructure can be very costly [29,50,51]. Additionally, man-made water systems such as those designed to handle sewer overflow may have negative impacts on water quality and animal health by association [52].

### Mosquito-targeting and currently available management models

The top ranked mosquito interventions, *larvicides* and modification *of man-made sites*, performed well on most “Public Health” Criteria. However, these interventions had economic, environmental and operational shortcomings that would need to be addressed in any comprehensive public health strategy. In the model examining only the list of currently available interventions, the rank ordering of interventions did not change for any of the six scenarios and included *larvicides*, *modification of man-made larval sites*, *status quo*, *use of parasites and pathogenic microorganisms* and *use of adulticides* in this order. This stable ranking across scenarios adds to the robustness of these interventions suggesting their capacity to meet current and higher intensity transmission scenario management demands.

### Combined model

In the combined model, four out of the top seven interventions included individual measures. This suggests that based on available evidence, current epidemiological levels of WNV, and values held by experts in Quebec, interventions aimed at personal level protection, source reduction or reduction of circulating levels of virus are most appropriate over habitat modification interventions and other forms of vector control and also under the higher transmission risk scenarios described in this study. These results are in agreement with the management options currently implemented in Quebec and elsewhere in North America although other forms of vector control (such as the use of adulticides) have been employed elsewhere in North America under high levels of WNV transmission [5,53].

### Limitations

It must be clarified that the MCDA approach is based on a socio-constructivist paradigm and that the validity of results are not based on strict reproducibility of results, but rather representativeness of society or relevant group of experts. The validity is also intimately tied to the coherence and transparency of results that are modeling a complex system. There are limits inherent in the choice of stakeholders, but the stakeholders chosen in our exercise were meant to be relevant to the dimensions at stake within the decision problem. In our example, as a first consultation, stakeholders from public health, wildlife and environmental management responded to our invitation to participate in this exercise. These stakeholders were representative of real-life management in the context of the study (small province where such files are managed by no more than 10–12 people) although many participants were indeed involved in previous WNV outbreaks. It is likely that given a different set of stakeholders, values expressed would be different.

With regards to interventions, from our initial stakeholder validated list, four interventions were found to currently lack sufficient data for evaluation (*use of lethal ovitraps*, *reduction in abundance of the main animal reservoir species*, *modification of habitat to reduce host reservoir species*, and *increasing biodiversity at the peridomestic level)*. While MCDA methods exist to deal with missing data [54], these were not explored in the current study to avoid speculating on their efficacy and acceptability. Future models should explore these interventions as data becomes available.

The exploration of multiple scenarios in the models did not yield very different rankings. While some differences in stakeholder weights were observed, convergence of stakeholder values was seen under scenarios of increased transmission severity; however, this did not strongly impact rankings. Many of the stakeholders have been working together on WNV related projects for a number of years which may in part explain the observed homogeneity in responses. A recommendation for future studies would be to include a more diverse group of stakeholders including, amongst others, front line clinicians responsible for providing care to the general population and members of the general population themselves to examine the potential variation in responses. Furthermore, to reduce workload, to explore low and high transmission scenarios first and if variations are found, to follow-up with medium transmission scenarios analyses where warranted.

Intervention evaluations were not re-assessed under the different scenarios. While many of these evaluations would likely not have changed, the social impact related evaluations might have with potential effects on rankings. However, no data were available to document this change for the current evaluation. An exploration of these and other potential changes to evaluations under different transmission scenarios in future studies may be warranted.

The PROMETHEE algorithm used in the ranking process provides a relative position for ordered interventions, therefore while general observations can be taken away from this analysis, such as individual preventive measures being preferred over regional-level interventions, the actual ranking results are valid only for the current model. In other words, middle or bottom ranked interventions should not necessarily be dismissed as being “poor”, rather they are less favoured over the top ranked interventions in the current model but still remain viable options to explore in future models or analyses as new options and information become available. Overall “poor” interventions, known to be so at the outset should not be included in the model in the first place. For this reason, it is worthwhile to explore specific subsets of interventions to further deepen our understanding of why one intervention may outperform another.

## Conclusions

While integrated vector management is often the primary recommendation for VBZD control [18,19,55], multicriteria decision analysis (MCDA) can be used to further refine the selection of complementary interventions for a VBZD management programme. MCDA can integrate cost-benefit analysis type information and other categories of concern including social acceptability and animal and environmental health concerns. Additionally, the use of scenarios enables the examination of tradeoffs between intervention performance and acceptability under different conditions.

The MCDA approach provides the opportunity to not only offer an informed recommendation to decision makers, but also an opportunity to build a shared understanding of the decision problem between different disciplines and sectors thereby increasing adhesion and support of all final recommendations. Further diversifying the stakeholder composition to include various representatives of society can also contribute to this process and should be explored in future projects.

Decisions are ultimately political but must be informed and supported by the best available evidence. While the explicit ranking of possible interventions often represent the main management objective driving a comparative assessment of interventions, the rigorous MCDA process in itself provides a framework to explicitly deconstruct stakeholder expressed priorities, rendering the decision-making process more transparent and arguably richer in its ability to document trade-offs and differing perspectives.

This project showed how a vector-borne and zoonotic diseases (VBZD) management model can be created to assess intervention options for the management of West Nile virus (WNV) at both the individual- and regional-levels taking into account currently available evidence now and under future potential transmission intensity scenarios, some of which are coherent with transmission intensity that may be seen under ongoing climate change. The results confirm that prevention of WNV via individual-level prevention measures such as well maintained window screens, in conjunction with source reduction regional-level interventions, such as *larvicides*, were top ranked interventions consistent with expressed stakeholders perspectives and in-line with currently stated WNV management objectives in the province of Quebec. Given the depth of both the model building exercise and broad similarities in approaching public health interventions for VBZD, we conclude that this current WNV model is likely useful as a base starting point for the analysis of other mosquito-borne diseases. Further work is warranted to better understand and clarify decision making mechanisms and determinants leading to selecting effective public health interventions for other VBZD now and under climate change.

## Supporting Information

S1 Appendix References used in the assessment of management interventions. (DOCX)

S1 Fig Additional individual-level protection strategy performance profiles. (TIF)

S2 Fig Regional-level management intervention profiles (interventions 11–16). (TIF)

S3 Fig Regional-level management intervention profiles (interventions 18–23). (TIF)

S1 Table Measurement scales used to score interventions in the model. (DOCX)

S2 Table Matrix of evaluation scores for the interventions in the Quebec WNV management model. (DOCX)

S3 Table Stakeholder weighting results by criteria and category for the Scenarios 1& 2 (low risk transmission). (DOCX)

S4 Table Stakeholder weighting results by criteria and category for the Scenarios 3& 4 (medium risk transmission). (DOCX)

S5 Table Stakeholder weighting results by criteria and category for the Scenarios 5& 6 (high risk transmission). (DOCX)
