# Supplementary material for: Assessing Interventions to Manage West Nile Virus Using Multi-Criteria Decision Analysis with Risk Scenarios
Source: PLoS One. 2016 Aug 5;11(8):e0160651. doi: 10.1371/journal.pone.0160651 (PMC4975439; doi:10.1371/journal.pone.0160651)
Supplement: S1 Appendix — (DOCX) [file pone.0160651.s001.docx]

**S1 Appendix. Supplemental References**

**Supporting references used in the assessment of management interventions**

1. Abramides, G.C., Roiz, D., Guitart, R., Quintana, S., Gimenez, N. (2013), Control of the Asian tiger mosquito (Aedes albopictus) in a firmly established area in Spain: risk factors and people's involvement, Transactions of The Royal Society of Tropical Medicine and Hygiene, 107(11), 706-714.

2. Adams, K. J., Chavasse, D. C., Mount, D. L., Carneiro, I. A., Curtis, C. F. (2002). Comparative insecticidal power of three pyrethroids on netting. Med Vet Entomol, 16(1), 106-108.

3. Alavi, M., Grebely, J., Matthews, G.V., Petoumenos, K., Yeung, B., Day, C., Lloyd, A.R., Van Beek, I., Kaldor, J.M., Hellard, M., Dore, G.J., Haber, P.S., the ATAHC Study Group (2012), Effect of pegylated interferon-+¦-2a treatment on mental health during recent hepatitis C virus infection, Journal of Gastroenterology and Hepatology, 27(5), 957-965.

4. Alphey, L. Alphey, N., 2014. Five things to know about genetically modified (GM) insects for vector control. PLoS pathogens, 10(3), p.e1003909.

5. Alphey, L., 2014. Genetic Control of Mosquitoes. Annual review of entomology, 59(1), p.205.

6. Amanna, I.J., Slifka, M.K. (2014), Current trends in West Nile virus vaccine development, Expert Rev Vaccines., 13(5), 589-608.

7. Andrade, C.F.S. Cabrini, I., (2010). Electronic mosquito repellers induce increased biting rates in Aedes aegypti mosquitoes (Diptera: Culicidae). Journal of vector ecology : journal of the Society for Vector Ecology, 35(1), pp.75–8.

8. Aquino, M., Fyfe, M., MacDougall, L., Remple, V. (2004). Protective behavior survey, West Nile virus, British Columbia. Emerg Infect Dis, 10(8), 1499-1501.

9. Bartlett-Healy, K., Hamilton, G., Healy, S., Crepeau, T., Unlu, I., Farajollahi, A., Fonseca, D., Gaugler, R., Clark, G.G., Strickman, D. (2011), Source reduction behavior as an independent measurement of the impact of a public health education campaign in an integrated vector management program for the Asian tiger mosquito, Int J Environ Res Public Health, 8(5), 1358-1367.

10. Basavaraju, S. V., Kuehnert, M. J., Zaki, S. R., Sejvar, J. J. (2014). Encephalitis caused by pathogens transmitted through organ transplants, United States, 2002-2013. Emerg Infect Dis, 20(9), 1443-1451.

11. Bellini, R., Zeller, H. Van Bortel, W., (2014). A review of the vector management methods to prevent and control outbreaks of West Nile virus infection and the challenge for Europe. Parasites & vectors, 7, p.323.

12. Benedict, M.Q. Robinson, A.S., (2003). The first releases of transgenic mosquitoes: an argument for the sterile insect technique. TRENDS in Parasitology, 19(8), pp.349–355.

13. Bennett, A.L., Smith, D.W., Cummins, M.J., Jacoby, P.A., Cummins, J.M., Beilharz, M.W. (2013), Low-dose oral interferon alpha as prophylaxis against viral respiratory illness: a double-blind, parallel controlled trial during an influenza pandemic year, Influenza. Other Respir Viruses., 7(5), 854-862.

14. Bill and Melinda Gates Foundation and Boston Consulting Group. (2007). Market Assessment for Public Health Pesticide Products. In B. C. Group (Ed.).

15. Blendon, R., NBenson, J., DesRoches, C., Hermann, M., Mackie, E., Weldon, K. (2003). Working Papers: Harvard School of Public Health Project on the Public and Biological Security and West Nile Virus Harvard School of Public Health.

16. Boisvert M. (2005). Suivi des populations de moustiques adultes dans des zones traitées et non traitées de la région métropolitaine de Montréal. Été 2004. Société de protection des forêts contre les insectes et maladies (SOPFIM).

17. Boisvert, M. (2005), Relevé des gîtes à Culex en milieu urbain, Société de protection des forêts contre les insectes et maladies (SOPFIM). 88p.

18. Bolduc D, Douville-Fradet M, Gingras D, Lambert L, Lavigne J, Pilon PA, Pinsonneault L, Samuel O (2006). Pertinence de maintenir l'application terrestre ou aérienne d'adulticides dans le plan d'intervention gouvernemental 2005. Institut national de santé publique du Québec, 13 p.

19. Bolduc, D., Côté R., Douville-Fradet M., Lambert, L., Pinsonneault, L. (2006). Le risque relié au virus du Nil occidental au Québec et les interventions à privilégier. Institut national de santé publique du Québec, 25p.

20. Bonds J.A.S. (2012). Ultra-low-volume space sprays in mosquito control: A critical review. Medical and Veritanary Entomology. Vol. 26. P. 121-130.

21. Boone, M. D. (2008). Examining the single and interactive effects of three insecticides on amphibian metamorphosis. Environ Toxicol Chem, 27(7), 1561-1568.

22. Bowen, R. A., Nemeth, N. M. (2007). Experimental infections with West Nile virus. Current opinion in infectious diseases, 20(3), 293-297.

23. Boyce W.M, Lawler S.P, Schultz J.M, McCauley S.J, Kimsey L.S, Niemela M.K, Nielsen C.F, Reisen W.K. (2007). Nontarget effects of the mosquito adulticide pyrethrin applied aerially during a West Nile virus outbreak in an urban California environment. J Am Mosq Control Assoc, 23(3):335-9.

24. Brander, S. M., Werner, I., White, J. W., Deanovic, L. A. (2009). Toxicity of a dissolved pyrethroid mixture to Hyalella azteca at environmentally relevant concentrations. Environ Toxicol Chem, 28(7), 1493-1499.

25. Brandler, S., Tangy, F. (2013), Vaccines in Development against West Nile Virus, Viruses, 5(10), 2384-2409.

26. Bukhari, T., Takken, W. Koenraadt, C.J., 2013. Biological tools for control of larval stages of malaria vectors-a review. Biocontrol Science and Technology, 23(9), pp.987–1023.

27. Carney, R.M. et al., 2008. Efficacy of aerial spraying of mosquito adulticide in reducing incidence of West Nile virus, California, 2005. Emerging Infectious Diseases, 14(5), p.747.

28. CDC. (2002). Update: Investigations of West Nile virus infections in recipients of organ transplantation and blood transfusion. MMWR Morb Mortal Wkly Rep, 51(37), 833-836.

29. CDC. (2004). Update: West Nile virus screening of blood donations and transfusion-associated transmission--United States, 2003. MMWR Morb Mortal Wkly Rep, 53(13), 281-284.

30. CDC. (2009). West Nile virus transmission via organ transplantation and blood transfusion - Louisiana, 2008. MMWR Morb Mortal Wkly Rep, 58(45), 1263-1267.

31. CDC. (2013). Fatal West Nile virus infection after probable transfusion-associated transmission--Colorado, 2012. MMWR Morb Mortal Wkly Rep, 62(31), 622-624.

32. CDC. (2003). Knowledge, attitudes, and behaviors about West Nile virus--Connecticut, 2002. MMWR. Morb Mortal Wkly Rep, 52(37), 886.

33. Chevalier P, St-Laurent L, Samuel O, Bolduc D. (2002). Larvicides pour contrer la transmission du virus du Nil accidental chez les humains. Institut national de santé publique du Québec. 46p.

34. Christophers, S. R. (1947). Mosquito repellents. Journal of Hygiene, 45(02), 176-231.

35. Clare, E.L. et al., (2011). Eating local: influences of habitat on the diet of little brown bats (Myotis lucifugus). Molecular ecology, 20(8), pp.1772–80.

36. Collier, B.W. et al., (2006). Field evaluation of mosquito control devices in southern Louisiana. Journal of the American Mosquito Control Association, 22(3), pp.444–50.

37. Colpitts, T.M., Conway, M.J., Montgomery, R.R., Fikrig, E. (2012), West Nile Virus: biology, transmission, and human infection, Clin Microbiol. Rev, 25(4), 635-648.

38. Conseil canadien des ministres de l’environnement. (2007). Recommandations canadiennes pour la qualité des eaux : protection de la vie aquatique – méthoprène. Dans Recommandations canadiennes pour la qualité de l’environnement, 1999, Conseil canadien des ministres de l’environnement, Winnipeg.

39. Croft, A. M. (2010). Malaria: prevention in travellers. Clin Evid (Online), 2010.

40. Custer, B., Busch, M. P., Marfin, A. A., Petersen, L. R. (2005). The cost-effectiveness of screening the U.S. blood supply for West Nile virus. Ann Intern Med, 143(7), 486-492.

41. Davis, R. S., Peterson, R. K. (2008). Effects of single and multiple applications of mosquito insecticides on nontarget arthropods. J Am Mosq Control Assoc, 24(2), 270-280.

42. Debboun, M., Strickman, D. (2013). Insect repellents and associated personal protection for a reduction in human disease. Med Vet Entomol, 27(1), 1-9.

43. DeGroote, J.P., Sugumaran, R. (2012), National and regional associations between human West Nile virus incidence and demographic, landscape, and land use conditions in the coterminous United States, Vector Borne Zoonotic Dis, 12(8), 657-665.

44. Delisle A, Davignon N, Sinaré S, Étude d’impact stratégique du Plan d’intervention gouvernemental de protection de la santé publique contre le virus du Nil occidental. Rapport sectoriel 6 : Profil social. Institut national de santé publique du Québec. 2005. 141 p.

45. DeLorenzo, M. E., Fulton, M. H. (2012). Comparative risk assessment of permethrin, chlorothalonil, and diuron to coastal aquatic species. Mar Pollut Bull, 64(7), 1291-1299.

46. Deparis, X., Frere, B., Lamizana, M., N'Guessan, R., Leroux, F., Lefevre, P., . . . Baudon, D. (2004). Efficacy of permethrin-treated uniforms in combination with DEET topical repellent for protection of French military troops in Cote d'Ivoire. J Med Entomol, 41(5), 914-921.

47. Des Lauriers A, Li J, Sze K, Baker S.L, Gris G, Chan J. A field study of the use of methoprene for West Nile Virus mosquito control. J. Environ. Eng. Sci. 5: 517-527. 2006.

48. Eamsila, C., Frances, S. P., Strickman, D. (1994). Evaluation of permethrin-treated military uniforms for personal protection against malaria in northeastern Thailand. J Am Mosq Control Assoc, 10(4), 515-521.

49. Elliott, S. J., Loeb, M., Harrington, D., Eyles, J. (2008). Heeding the message? Determinants of risk behaviours for West Nile virus. Can J Public Health, 99(2), 137-141.

50. Enayati, A., Hemingway, J., Garner, P. (2007). Electronic mosquito repellents for preventing mosquito bites and malaria infection. Cochrane Database Syst Rev, 2.

51. Erlanger, T.E., Keiser, J., Utzinger, J. (2008), Effectiveness of dengue vector control in developing countries: systematic literature review and meta-analysis, Impact on Health Caused by Water Resources Development and Management Projects and Health Impact Assessment as a Tool for Mitigation, 22 p. 149.

52. Faulde, M., Uedelhoven, W. (2006). A new clothing impregnation method for personal protection against ticks and biting insects. Int J Med Microbiol, 296 Suppl 40, 225-229.

53. Faulde, M., Albiez, G., Nehring, O. (2012). Novel long-lasting impregnation technique transferred from clothing to bednets: extended efficacy and residual activity of different pyrethroids against Aedes aegypti as shown by EN ISO 6330-standardized machine laundering. Parasitol Res, 110(6), 2341-2350. doi: 10.1007/s00436-011-2769-6

54. Gemmell, N.J., Jalilzadeh, A., Didham, R.K., Soboleva, T., Tompkins, D.M. (2013), The Trojan female technique: a novel, effective and humane approach for pest population control, Proceedings of the Royal Society B: Biological Sciences, 280(1773).

55. Geraghty E.M, Margolis H.G, Kjemtrup A, Reisen W, Franks P. Correlation between aerial insecticide spraying to interrupt West Nile Virus transmission and emergency Department visit ion Sacremento County, California. Public Health Report. Vol. 128, 2013. p.221-230.

56. Gibney, K.B., Colborn, J., Baty, S., Bunko Patterson, A.M., Sylvester, T., Briggs, G., Stewart, T., Levy, C., Komatsu, K., MacMillan, K., Delorey, M.J., Mutebi, J.P., Fischer, M., Staples, J.E. (2012), Modifiable risk factors for West Nile virus infection during an outbreak--Arizona, 2010, Am J Trop. Med Hyg, 86(5), 895-901.

57. Goodman, R.A., Buehler, J.W. (2009), Delinquent mortgages, neglected swimming pools, and West Nile virus, California, Emerg. Infect Dis, 15(3), 508-509.

58. Goodyer, L.I., Croft, A.M., Frances, S.P., Hill, N., Moore, S.J., Onyango, S.P., Debboun, M. (2010), Expert Review of the Evidence Base for Arthropod Bite Avoidance, Journal of Travel Medicine, 17(3), 182-192.

59. Gopalakrishnan, R., Chaurasia, A.K., Baruah, I., Veer, V. (2013), Evaluation of permethrin-impregnated military uniforms for contact toxicity against mosquitoes and persistence in repeated washings, Int. J. Environ. Sci. Technol., 1-6.

60. Grazzini, G., Liumbruno, G. M., Pupella, S., Silvestri, A. R., Randi, V., Pascarelli, N., . . . Sambri, V. (2008). West Nile virus in Italy: a further threat to blood safety, a further challenge to the blood system. Blood Transfus, 6(4), 235-237.

61. Grondin J, Corriveau R, Bolduc D et Brunelle M. (2003). Virus du nil occidental : Évaluation des attitudes, Des comportements et des Connaissances populaires. Institut national de santé publique. 69p.

62. Gujral, I. B., Zielinski-Gutierrez, E. C., LeBailly, A., Nasci, R. (2007). Behavioral risks for West Nile virus disease, northern Colorado, 2003. Emerg Infect Dis, 13(3), 419-425.

63. Gupta, R. K., Rutledge, L. C., Reifenrath, W. G., Gutierrez, G. A., Korte, D. W., Jr. (1989). Effects of weathering on fabrics treated with permethrin for protection against mosquitoes. J Am Mosq Control Assoc, 5(2), 176-179.

64. Gupta, R. K., Sweeney, A. W., Rutledge, L. C., Cooper, R. D., Frances, S. P., Westrom, D. R. (1987). Effectiveness of controlled-release personal-use arthropod repellents and permethrin-impregnated clothing in the field. J Am Mosq Control Assoc, 3(4), 556-560.

65. Harbach, R. E., Tang, D. B., Wirtz, R. A., Gingrich, J. B. (1990). Relative repellency of two formulations of N,N-diethyl-3-methylbenzamide (deet) and permethrin-treated clothing against Culex sitiens and Aedes vigilax in Thailand. J Am Mosq Control Assoc, 6(4), 641-644.

66. Harbison, J.E., Metzger, M.E., Hu, R. (2010), Association between Culex quinquefasciatus (Diptera: Culicidae) oviposition and structural features of belowground stormwater treatment devices, J Med Entomol., 47(1), 67-73.

67. Harley, D., Ritchie, S., Bain, C., Sleigh, A. C. (2005). Risks for Ross River virus disease in tropical Australia. International Journal of Epidemiology, 34(3), 548-555.

68. Henderson, J. P. W. R. G. T. (2006). An Assessment of the Effectiveness of the Mosquito Magnet Pro Model for Suppression of Nuisance Mosquitoes. J Am Mosq Control Assoc Journal of the American Mosquito Control Association, 22(3), 401-407.

69. Herrington, J. E., Jr. (2003). Pre-West Nile virus outbreak: perceptions and practices to prevent mosquito bites and viral encephalitis in the United States. Vector Borne Zoonotic Dis, 3(4), 157-173.

70. Hribar LJ, Fussell EM, Leal AL. Larviciding offshore islands reduces adulticidal treatment of populated areas adjacent to national wildlife refuges. J Am Mosq Control Assoc; 2011. Dec;27(4):408-13.

71. Hu X, Liu Y, Wu J. Culling structured hosts to eradicate vector-borne diseases. Math Biosci Eng. 2009 Apr;6(2):301-19.

72. Iyer, A.V., Kousoulas, K.G. (2013), A Review of Vaccine Approaches for West Nile Virus, Int. J. Environ. Res. Public Health, 10(9), 4200-4233.

73. Jackson, M.J. et al., 2012. An Evaluation of the Effectiveness of a Commercial Mechanical Trap to Reduce Abundance of Adult Nuisance Mosquito Populations. Journal of the American Mosquito Control Association, 28(4), pp.292–300.

74. Jackson, M.J. et al., 2013. Modelling factors that affect the presence of larval mosquitoes (Diptera: Culicidae) in stormwater drainage systems to improve the efficacy of control programmes. The Canadian Entomologist, 145(06), pp.674–685.

75. Katz, T. M. J. H. A. (2008). Insect repellents: Historical perspectives and new developments. Journal of the American Academy of Dermatology Journal of the American Academy of Dermatology, 58(5), 865-871.

76. Kiberd, B. A., Forward, K. (2004). Screening for West Nile virus in organ transplantation: a medical decision analysis. Am J Transplant, 4(8), 1296-1301

77. Kilpatrick, A.M., Dupuis, A.P., Chang, G.J., Kramer, L.D. (2010), DNA vaccination of American robins (Turdus migratorius) against West Nile virus, Vector Borne Zoonotic Dis, 10(4), 377-380.

78. Kimani, E. W., Vulule, J. M., Kuria, I. W., Mugisha, F. (2006). Use of insecticide-treated clothes for personal protection against malaria: a community trial. Malar J, 5, 63.

79. Kleinman, S. H., Williams, J. D., Robertson, G., Caglioti, S., Williams, R. C., Spizman, R., . . . Busch, M. P. (2009). West Nile virus testing experience in 2007: evaluation of different criteria for triggering individual-donation nucleic acid testing. Transfusion, 49(6), 1160-1170.

80. Kline, D.L., (2006). Traps and trapping techniques for adult mosquito control. Journal of the American Mosquito Control Association, 22(3), pp.490–496.

81. Koren, G., Matsui, D., Bailey, B. (2003). DEET-based insect repellents: safety implications for children and pregnant and lactating women. CMAJ, 169(3), 209-212.

82. Labbé, Y., Aubé-Maurice, B., Vézina, A., Boivert, J., Gingras, D. (2006), Revue des mesures de prévention et de protection contre le virus du Nil occidental. Étude d'impact stratégique du Plan d'intervention gouvernemental de protection de la santé publique contre le virus du Nil occudental. Rapport sectoriel 3, Institut national de santé publique du Québec, 3, 143 p.

83. LaBeaud, A. D., Kile, J. R., Kippes, C., King, C. H., Mandalakas, A. M. (2007). Exposure to West Nile virus during the 2002 epidemic in Cuyahoga County, Ohio: a comparison of pediatric and adult behaviors. Public Health Rep, 122(3), 356-361.

84. LaBeaud, A. D., Lisgaris, M. V., King, C. H., Mandalakas, A. M. (2006). Pediatric West Nile virus infection: neurologic disease presentations during the 2002 epidemic in Cuyahoga County, Ohio. Pediatr Infect Dis J, 25(8), 751-753

85. Laliberté, C., Hubert, B., Corriveau, R., Farley, C., Bolduc, D., Lavigne, J., Pilon, P.A., Lambert, L. (2005), Mesures individuelles et collectives pour prévenir la transmission du virus du Nil occidental - Éléments pour un plan global d'intervention., Institut national de santé publique du Québec, Québec, 69 p.

86. Lalone, C. A., Villeneuve, D. L., Burgoon, L. D., Russom, C. L., Helgen, H. W., Berninger, J. P., . . . Ankley, G. T. (2013). Molecular target sequence similarity as a basis for species extrapolation to assess the ecological risk of chemicals with known modes of action. Aquat Toxicol, 144-145, 141-154.

87. Lawler, S.P., Reimer, L., Thiemann, T., Fritz, J., Parise, K., Feliz, D., Elnaiem, D.E. (2007), Effects of vegetation control on mosquitoes in seasonal freshwater wetlands, J Am Mosq. Control Assoc, 23(1), 66-70.

88. Lee, B. Y., Biggerstaff, B. J. (2006). Screening the United States blood supply for West Nile Virus: a question of blood, dollars, and sense. PLoS Med, 3(2), e99.

89. Li J.Y, Chin L, Luciani P.D, Des Lauriers A, , Sze K, Shao J, Komer W, Wilkinson K, Truen D, Anderton R. Environmental factors affecting methoprene concentrations for West Nile Virus in a storm sewer system. Water Qual. R. J Can. 44(2)TBA. 2009.

90. Liu, W., Zhang, J., Hashim, J. H., Jalaludin, J., Hashim, Z., Goldstein, B. D. (2003). Mosquito coil emissions and health implications. Environmental health perspectives, 111(12), 1454.

91. Loeb, M., Elliott, S. J., Gibson, B., Fearon, M., Nosal, R., Drebot, M., . . . Eyles, J. (2005). Protective behavior and West Nile virus risk. Emerg Infect Dis, 11(9), 1433-1436.

92. Lothrop H.D, Lothrop B.B, Gomsi D.E, Reisen W.K. (2008). Intensive Early Season Adulticide Applications Decrease Arbovirus Transmission Throughout the Coachella Valley, Riverside County, California. Vector-borne and zoonotic diseases. Volume 8, Number 4,. P. 475-489.

93. Lund, A., McMillan, J., Kelly, R., Jabbarzadeh, S., Mead, D.G., Burkot, T.R., Kitron, U., Vazquez-Prokopec, G.M. (2014), Long term impacts of combined sewer overflow remediation on water quality and population dynamics of Culex quinquefasciatus, the main urban West Nile virus vector in Atlanta, GA, Environ Res, 129 20-26.

94. Majambere, S., Massue, D. J., Mlacha, Y., Govella, N. J., Magesa, S. M., Killeen, G. F. (2013). Advantages and limitations of commercially available electrocuting grids for studying mosquito behaviour. Parasit Vectors, 6, 53.

95. McCarthy, T. A., Hadler, J. L., Julian, K., Walsh, S. J., Biggerstaff, B. J., Hinten, S. R., . . . Petersen, L. R. (2001). West Nile virus serosurvey and assessment of personal prevention efforts in an area with intense epizootic activity: Connecticut, 2000. Ann N Y Acad Sci, 951, 307-316.

96. McGraw, E.A., O'Neill, S.L. (2013), Beyond insecticides: new thinking on an ancient problem, Nat Rev Micro, 11(3), 181-193.

97. Micieli, M.V., Glaser, R.L. (2014), Somatic wolbachia (Rickettsiales: Rickettsiaceae) levels in culex quinquefasciatus and culex pipiens (diptera: Culicidae) and resistance to west Nile virus infection, Journal of Medical Entomology, 51(1), 189-199.

98. Mickle R.E., Samuel O., St-Laurent L., Dumas P. et G. Rousseau. Direct comparison of deposit from aerial and ground ULV applications of malathion with AGDISP predictions. REMSpC Counsulting, Direction de la toxicologie humaine/Institut national de santé publique du Québec, Société de protection des forêts contre les insectes et maladies. 74 pages. 2005.

99. Micucci S. (2004). The effectiveness of methoprene for controlling mosquito populations in Ontario that can carry West Nile virus (Provisional abstract). Database of Abstracts of Reviews of Effects; 95.

100. Morse, D. L. (2003). West Nile virus--not a passing phenomenon. N Engl J Med, 348(22), 2173-2174.

101. MSSS (2007) La santé, autrement dit... Pour espérer vivre plus longtemps et en meilleure santé. http://msssa4.msss.gouv.qc.ca/fr/document/publication.nsf/0/1a165acb041a1e7a852572db004c26f3?OpenDocument

102. MSSS (2013). Se protéger du soleil et des rayons UV. Disponible au site http://www.sante.gouv.qc.ca/conseils-et-prevention/se-proteger-du-soleil-et-des-rayons-uv/?utm_expid=57681679-0.Q8XbASQ8QWuE8joG7STJnw.0&utm_referrer=http%3A%2F%2Fwww.msss.gouv.qc.ca%2Fsujets%2Fsantepub%2Fenvironnement%2Findex.php%3Fsoleil-et-rayons-uv

103. Nett, R. J., Kuehnert, M. J., Ison, M. G., Orlowski, J. P., Fischer, M., Staples, J. E. (2012). Current practices and evaluation of screening solid organ donors for West Nile virus. Transpl Infect Dis, 14(3), 268-277. doi: 10.1111/j.1399-3062.2012.00743.x

104. Niranjan Reddy, B.P., Gupta, B., Rao, B.P. (2014), Vector population manipulation for control of arboviruses-a novel prospect for India, Pest Management Science, 70(4), 517-523.

105. Nolan, M.S., Zangeneh, A., Khuwaja, S.A., Martinez, D., Rossmann, S.N., Cardenas, V., Murray, K.O. (2012), Proximity of Residence to Bodies of Water and Risk for West Nile Virus Infection: A Case-Control Study in Houston, Texas, BioMed Research International.

106. O'Brien, S. F., Scalia, V., Zuber, E., Hawes, G., Alport, E. C., Goldman, M., Fearon, M. A. (2010). West Nile virus in 2006 and 2007: the Canadian Blood Services' experience. Transfusion, 50(5), 1118-1125.

107. Ogoma, S. B., Moore, S. J., Maia, M. F. (2012). A systematic review of mosquito coils and passive emanators: defining recommendations for spatial repellency testing methodologies. Parasit Vectors, 5, 287.

108. OMS. World Health Organisation. Baccillus Thuringiencis. Environmental Health Criteria 217, International Programme on Chemical Safety. 1999; 105 p.

109. Ozdenerol, E., Bialkowska-Jelinska, E., Taff, G.N. (2008), Locating suitable habitats for West Nile Virus-infected mosquitoes through association of environmental characteristics with infected mosquito locations: a case study in Shelby County, Tennessee, Int J Health Geogr, 7 p. 12.

110. Palmisano CT, Taylor V, Caillouet K, Byrd B, Wesson DM (2005). Impact of West Nile virus outbreak upon St. Tammany Parish Mosquito Abatement District. J Am Mosq Control Assoc, 21(1):33-8.

111. Parent, L. M., Delorenzo, M. E., Fulton, M. H. (2011). Effects of the synthetic pyrethroid insecticide, permethrin, on two estuarine fish species. J Environ Sci Health B, 46(7), 615-622.

112. Patterson, R.S., Weidhaas, D.E., Ford, H.R., Lofgren, C.S. (1970), Suppression and Elimination of an Island Population of Culex pipiens quinquefasciatus with Sterile Males, Science, 168(3937), 1368-1369.

113. Pauluhn, J. M. U. (2006). Mosquito coil smoke inhalation toxicity. Part I: Validation of test approach and acute inhalation toxicity. Journal of Applied Toxicology, 26(3), 269-278.

114. Pauluhn, J. M. U. (2006). Mosquito coil smoke inhalation toxicity. Part II: Subchronic nose-only inhalation study in rats. Journal of Applied Toxicology, 26(3), 279-292.

115. Petersen, L.R., Brault, A.C., Nasci, R.S. (2013), West Nile Virus: Review of the Literature, JAMA, 310(3), 308-315.

116. Peterson R K.D, Macedo P.A, Davis R.S. A Human-Health Risk Assessment for West Nile Virus and Insecticides Used in Mosquito Management. Environ Health Perspect. Mar 2006; 114(3): 366–372.

117. Phillips, B. M., Anderson, B. S., Voorhees, J. P., Siegler, K., Denton, D., Tenbrook, P., . . . Tjeerdema, R. S. (2014). Monitoring the aquatic toxicity of mosquito vector control spray pesticides to freshwater receiving waters. Integr Environ Assess Manag.

118. Pinsonneault L, Niyonsenga T, Lebel G. Mosquito control and corvidea surveillance: exploring spatial variations in mosquito abundance and corvidea counts in the province of Québec 2002-2003 (poster). San Francisco. 2006.

119. Pupella, S., Pisani, G., Cristiano, K., Catalano, L., Grazzini, G. (2013). West Nile virus in the transfusion setting with a special focus on Italian preventive measures adopted in 2008-2012 and their impact on blood safety. Blood Transfus, 11(4), 563-574.

120. Pupella, S., Pisani, G., Cristiano, K., Catalano, L., Grazzini, G. (2014). Update on West Nile virus in Italy. Blood Transfus, 12(4), 626-627.

121. Rabe, I. B., Schwartz, B. S., Farnon, E. C., Josephson, S. A., Webber, A. B., Roberts, J. P., . . . Glaser, C. A. (2013). Fatal transplant-associated west nile virus encephalitis and public health investigation-california, 2010. Transplantation, 96(5), 463-468.

122. Ratterree, M. S., Gutierrez, R. A., Travassos da Rosa, A. P., Dille, B. J., Beasley, D. W., Bohm, R. P., . . . Tesh, R. B. (2004). Experimental infection of rhesus macaques with West Nile virus: level and duration of viremia and kinetics of the antibody response after infection. J Infect Dis, 189(4), 669-676.

123. Ravel-Nelson, P., Soin, K., Tolerud, S. (2005). Analysis of Bacillus sphaericus in controlling mosquito populations in urban catch basins. Journal of environmental health. 67(7): 28: 28-31.

124. Reddy M.R. Spielman A, Lepore T.J, Henley D, Kiszewski A.E, Reiter P. (2006). Efficacy of resmethrin aerosols applied from the road for suppressing Culex vectors of West Nile virus. Vector Borne Zoonotic Dis, 6(2):117-27.

125. Reisen W, Brault AC (2007). West Nile virus in North America: perspectives on epidemiology and intervention. Pest Manag Sci, Vol. 63, No 7, pp. 641-6.

126. Reisen, W.K., Takahashi, R.M., Carroll, B.D., Quiring, R. (2008), Delinquent mortgages, neglected swimming pools, and West Nile virus, California, Emerg Infect Dis, 14(11), 1747-1749.

127. Rey, J.R., Walton, W.E., Wolfe, R.J., Connelly, C.R., O'Connell, S.M., Berg, J., Sakolsky-Hoopes, G.E., Laderman, A.D. (2012), North American wetlands and mosquito control, Int J Environ Res Public Health, 9(12), 4537-4605.

128. Rowland, M., Durrani, N., Hewitt, S., Mohammed, N., Bouma, M., Carneiro, I., Rozendaal, J., Schapira, A. (1999). Permethrin-treated chaddars and top-sheets: appropriate technology for protection against malaria in Afghanistan and other complex emergencies. Trans R Soc Trop Med Hyg, 93(5), 465-472.

129. Ruktanonchai, D.J., Stonecipher, S., Lindsey, N., McAllister, J., Pillai, S.K., Horiuchi, K., Delorey, M., Biggerstaff, B.J., Sidwa, T., Zoretic, J., Nasci, R., Fischer, M., Hills, S.L. (2014). Effect of aerial insecticide spraying on West Nile virus disease-north-central Texas, 2012. The American journal of tropical medicine and hygiene, 91(2), pp.240–5.

130. Schleier, J. J., Peterson, R. K. (2013). A refined aquatic ecological risk assessment for a pyrethroid insecticide used for adult mosquito management. Environ Toxicol Chem, 32(4), 948-953.

131. Schofield, S., Crane, F., Tepper, M. (2012). Good interventions that few use: uptake of insect bite precautions in a group of Canadian Forces personnel deployed to Kabul, Afghanistan. Mil Med, 177(2), 209-215.

132. Schreck, C. E., Posey, K., Smith, D. (1978). Durability of permethrin as a potential clothing treatment to protect against blood-feeding arthropods. J Econ Entomol, 71(3), 397-400.

133. Shelley, L. K., Ross, P. S., Kennedy, C. J. (2012). Immunotoxic and cytotoxic effects of atrazine, permethrin and piperonyl butoxide to rainbow trout following in vitro exposure. Fish Shellfish Immunol, 33(2), 455-458.

134. Sholdt, L. L., Schreck, C. E., Qureshi, A., Mammino, S., Aziz, A., Iqbal, M. (1988). Field bioassays of permethrin-treated uniforms and a new extended duration repellent against mosquitoes in Pakistan. J Am Mosq Control Assoc, 4(3), 233-236.

135. Simpson, J.E., Hurtado, P.J., Medlock, J., Molaei, G., Andreadis, T.G., Galvani, A.P., uk-Wasser, M.A. (2012), Vector host-feeding preferences drive transmission of multi-host pathogens: West Nile virus as a model system, Proceedings of the Royal Society B: Biological Sciences, 279(1730), 925-933.

136. Smith, J.P., Cope, E.H., Walsh, J.D., Hendrickson, C.D. (2010). Ineffectiveness of mass trapping for mosquito control in St. Andrews State Park, Panama City Beach, Florida. Journal of the American Mosquito Control Association, 26(1), pp.43–9.

137. Soto, J., Medina, F., Dember, N., Berman, J. (1995). Efficacy of permethrin-impregnated uniforms in the prevention of malaria and leishmaniasis in Colombian soldiers. Clin Infect Dis, 21(3), 599-602.

138. Stockwell P.J, Wessell N, Reed D.R, Kronenwetter-Koepel T.A, Reed K.T, Turchi T.R, Meece J.K. (2006). A field evaluation of four larval mosquito control methods in urban catch basins. Journal of the mosquito Control Association. 22(4):666-671.

139. Sudakin, D. L., Trevathan, W. R. (2003). DEET: a review and update of safety and risk in the general population. J Toxicol Clin Toxicol, 41(6), 831-839.

140. Sukumaran, D., Sharma, A.K., Wasu, Y.H., Pandey, P., Tyagi, V. (2014). Knockdown and repellent effect of permethrin-impregnated army uniform cloth against Aedes aegypti after different cycles of washings. Parasitology research, 113(5), pp.1739–47.

141. Surgeoner, G., Helson, B. (1978). A field evaluation of electrocutors for mosquito control in southern Ontario. Paper presented at the Proceedings of the Entomological Society of Ontario.

142. Tedesco C, Ruiz M, McLafferty S (2010). Mosquito politics: local vector control policies and the spread of West Nile Virus in the Chicago region. Health Place, Vol. 16, No. 6, pp.1188-95.

143. Tilley, P. A., Fox, J. D., Lee, B., Chui, L., Preiksaitis, J. (2008). Screening of organ and tissue donors for West Nile virus by nucleic acid amplification--a three year experience in Alberta. Am J Transplant, 8(10), 2119-2125.

144. Trudel R, Leclerc L, Souto-Neveu M. (2013). Rapport des travaux d'application de larvicides en prévention (saison 2013): volet contrôle vectoriel du plan d'intervention gouvernemental contre le virus du Nil occidental. Société de protection des forêts contre les insectes et les maladies.

145. Tuiten, W., Koenraadt, C.J., McComas, K., Harrington, L.C. (2009), The effect of West Nile virus perceptions and knowledge on protective behavior and mosquito breeding in residential yards in upstate New York, Ecohealth, 6(1), 42-51.

146. Tusting, L.S., Thwing, J., Sinclair, D., Fillinger, U., Gimnig, J., Bonner, K.E., Bottomley, C., Lindsay, S.W. (2013), Mosquito larval source management for controlling malaria [Systematic Review], Cochrane Database of Systematic Reviews 2013;(8),(8).

147. U.S. Environmental Protection Agency. (2006). Permethrin Facts (Reregistration Eligibility Decision (RED) Fact Sheet). http://www.epa.gov/pesticides/reregistration/REDs/factsheets/permethrin_fs.htm

148. Valcke M, Belleville D. Évaluation des risques toxicologiques associés à l’utilisation d’adulticides dans le cadre d’un programme de lutte vectorielle contre la transmission du virus du Nil occidental. Direction des risques biologiques, environnementaux et occupationnels. Institut national de santé publique du Québec. 2002. 89 pages. Annexes.

149. Vazquez-Prokopec, G.M., Eng, J.L.V., Kelly, R., Mead, D.G., Kolhe, P., Howgate, J., Kitron, U., Burkot, T.R. (2010), The risk of West Nile virus infection is associated with combined sewer overflow streams in urban Atlanta, Georgia, USA, Environ Health Perspect, 118(10), p. 1382.

150. Weigel, S., Kuhlmann, J., Huhnerfuss, H. (2002). Drugs and personal care products as ubiquitous pollutants: occurrence and distribution of clofibric acid, caffeine and DEET in the North Sea. Sci Total Environ, 295(1-3), 131-141.

151. Whitaker Jr, J.O., 2004. Prey selection in a temperate zone insectivorous bat community. Journal of Mammalogy, 85(3), pp.460–469.

152. Wilson, S. D., Varia, M., Lior, L. Y., Field Epidemiology Summer Course. (2005). West Nile Virus: the buzz on Ottawa residents' awareness, attitudes and practices. Can J Public Health, 96(2), 109-113.

153. Winston, D. J., Vikram, H. R., Rabe, I. B., Dhillon, G., Mulligan, D., Hong, J.C., Busuttil, R.W., Nowicki, M.J., Mone, T., Civen, R., Tecle, S.A., Trivedi, K.K., Hocevar, S.N.; West Nile Virus Transplant-Associated Transmission Investigation Team. (2014). Donor-derived West Nile virus infection in solid organ transplant recipients: report of four additional cases and review of clinical, diagnostic, and therapeutic features. Transplantation, 97(9), 881-889.

154. Wolf, R. F., Papin, J. F., Hines-Boykin, R., Chavez-Suarez, M., White, G. L., Sakalian, M., Dittmer, D. P. (2006). Baboon model for West Nile virus infection and vaccine evaluation. Virology, 355(1), 44-51.

155. Yang, Y., Ma, H., Zhou, J., Liu, J., Liu, W. (2014). Joint toxicity of permethrin and cypermethrin at sublethal concentrations to the embryo-larval zebrafish. Chemosphere, 96, 146-154.

156. Yango, A. F., Fischbach, B. V., Levy, M., Chandrakantan, A., Tan, V., Spak, C., Melton, L., Rice, K., Barri, Y., Rajagopal, A., Klintmalm, G. (2014). West Nile virus infection in kidney and pancreas transplant recipients in the Dallas-Fort Worth Metroplex during the 2012 Texas epidemic. Transplantation, 97(9), 953-957.

157. Young, J.A., Jefferies, W. (2013), Towards the Conservation of Endangered Avian Species: A Recombinant West Nile Virus Vaccine Results in Increased Humoral and Cellular Immune Responses in Japanese Quail Coturnix japonica, PLoS One, 8(6), p. e67137.
