# Supplementary figures and images for: Assessing Interventions to Manage West Nile Virus Using Multi-Criteria Decision Analysis with Risk Scenarios
Source: PLoS One. 2016 Aug 5;11(8):e0160651. doi: 10.1371/journal.pone.0160651 (PMC4975439; doi:10.1371/journal.pone.0160651)

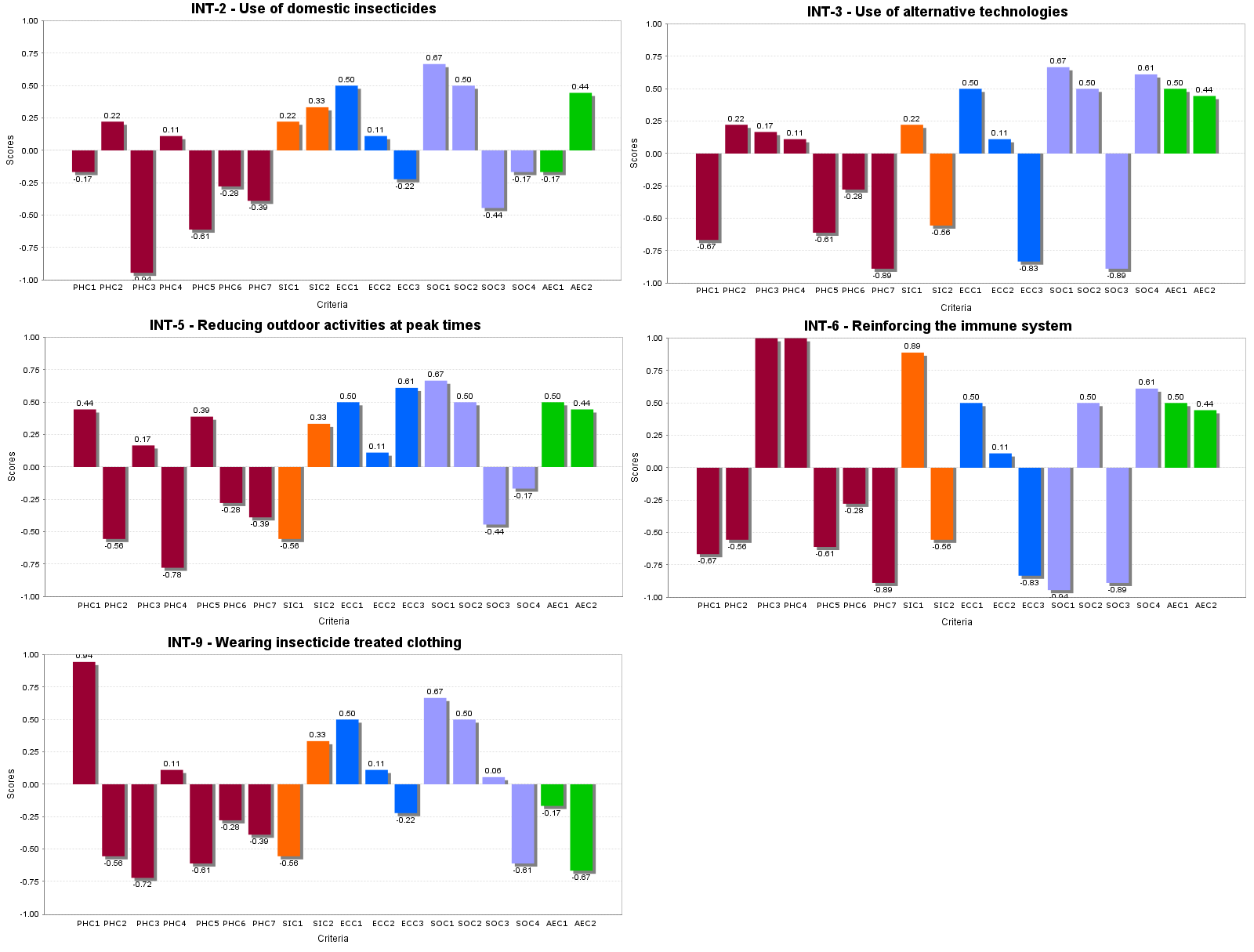

Supplement: S1 Fig — (TIF) [file pone.0160651.s002.tif]

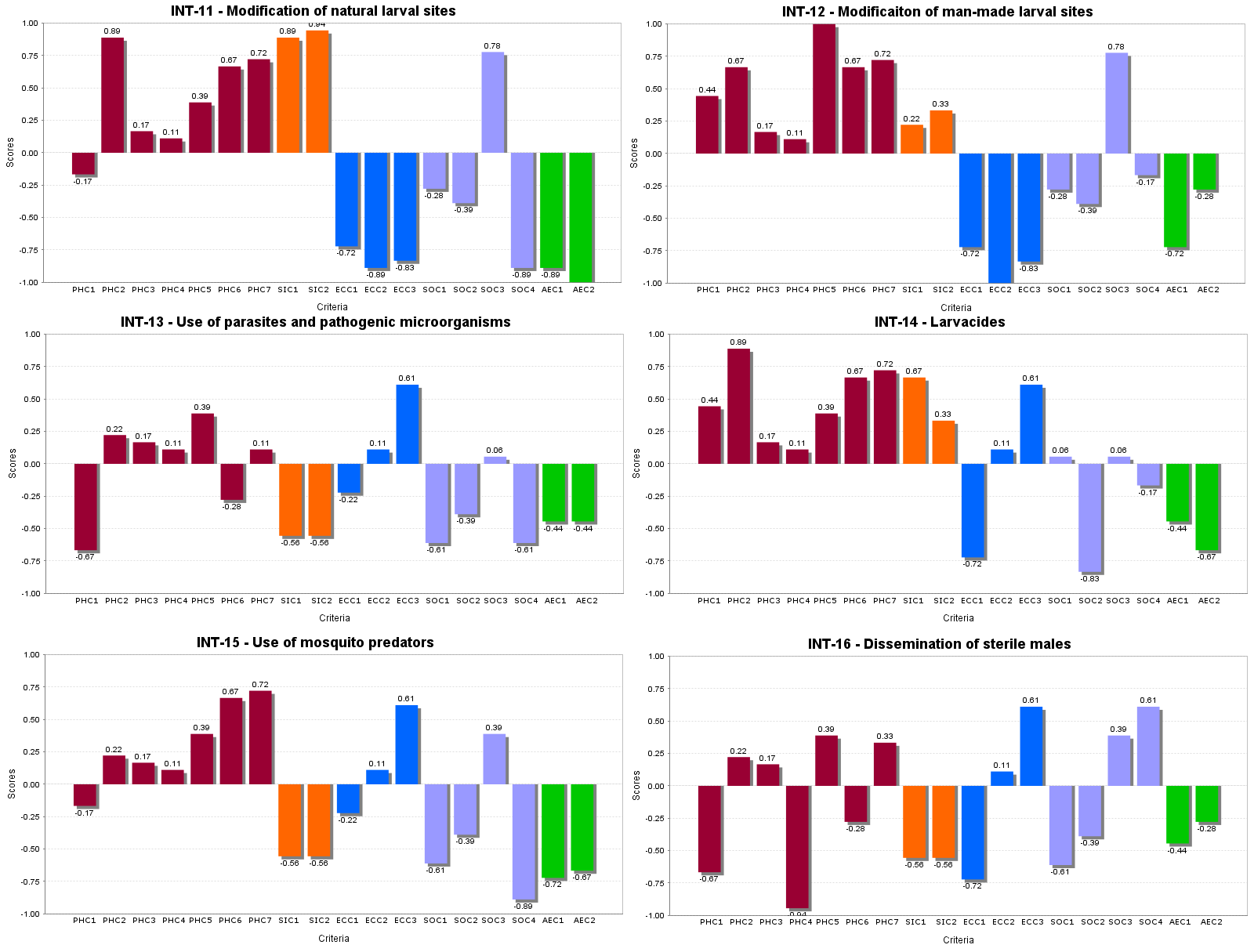

Supplement: S2 Fig — (TIF) [file pone.0160651.s003.tif]

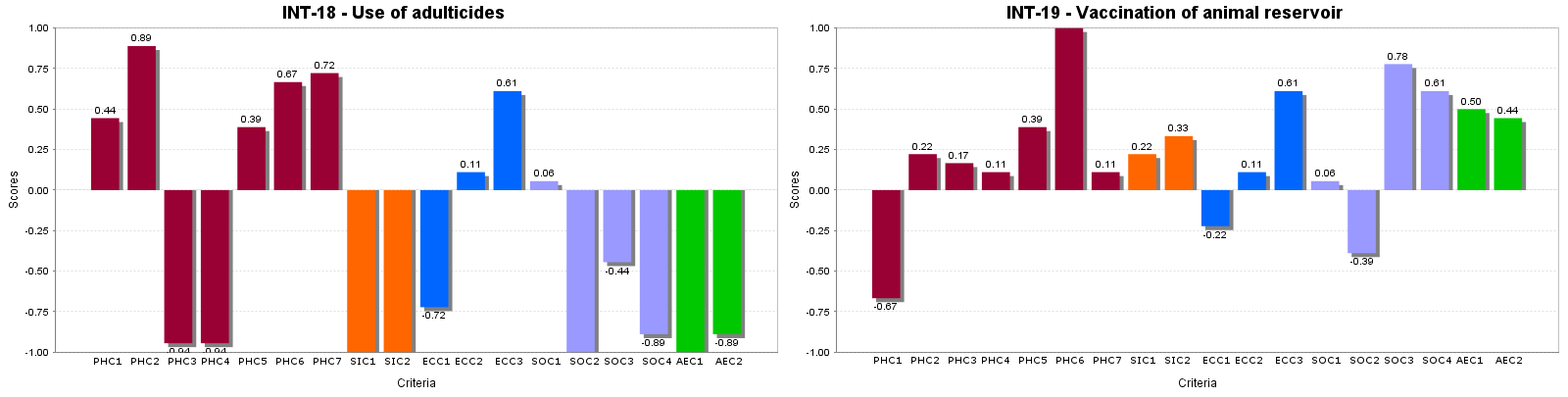

Supplement: S3 Fig — (TIF) [file pone.0160651.s004.tif]
