# Supplementary material for: Assessing Interventions to Manage West Nile Virus Using Multi-Criteria Decision Analysis with Risk Scenarios
Source: PLoS One. 2016 Aug 5;11(8):e0160651. doi: 10.1371/journal.pone.0160651 (PMC4975439; doi:10.1371/journal.pone.0160651)
Supplement: S1 Table — (DOCX) [file pone.0160651.s005.docx]

**S1 Table. Measurement scales used to score interventions in the model**

| **Category** | **WNV criteria** | **Description** | **Measurement scale** | | |
| --- | --- | --- | --- | --- | --- |
| **Public Health Criteria (PHC)** | | | | | |
|  | PHC1 - Incidence reduction | Reduction in incidence of human cases (or case proportion in population) | -1 : increase in cases  0: no difference  1: small reduction  2: moderate reduction  3: high reduction  4: significant reduction | | |
|  | PHC2 - Entomological risk reduction | Reduction of entomological risk (infection rate or abundance of main vectors) | -1 : increased risk  0: no difference  1: small reduction  2: moderate reduction  3: high reduction  4: significant reduction | | |
|  | PHC3 –Physical health impact | Impacts to human physical health (including susceptible populations)  **= Intensity * Duration of Effect** | **Intensity**:  • -4 : significant beneficial effect  • -3 : high beneficial effect  • -2: moderate beneficial effect  • -1: minimal beneficial effect  • 0: no effect  • 1: minimal adverse and reversible effect  • 2: moderate adverse effect  • 3: high adverse effect  • 4: significant adverse effect | | **Duration of effect** :  • 1: short -term effect, reversible  • 2 short-term effect, reversible  • 3: sub- chronic effect, reversible  • 4: chronic effect, irreversible |
|  | PHC4 - Mental health impact | Impacts to human mental and psychosocial health (including susceptible populations)  **= Intensity * Duration of Effect** | **Intensity**:  • -4 : significant beneficial effect  • -3 : High beneficial effect  • -2: moderate beneficial effect  • -1: minimal beneficial effect  • 0: no effect  • 1: minimal adverse and reversible effect  • 2: moderate adverse effect  • 3: high adverse effect  • 4: significant adverse effect | | **Duration of effect** :  • 1: short -term effect, reversible  • 2 short-term effect, reversible  • 3: sub- chronic effect, reversible  • 4: chronic effect, irreversible |
|  | PHC5 – Social equity | Impact on social equity | • -2: very positive effect  • -1: rather positive effect  • 0: no effect  • 1: rather negative effect  • 2: very negative effect | | |
|  | PHC6 – Reduction of circulating virus | Reduction in level of circulating virus in animal reservoir (infection rate or density of population) | • 1: increase  • 0: no difference  • 1: small reduction  • 2: moderate reduction  • 3: High reduction  • 4: significant reduction | | |
|  | PHC7 – Proportion affected | Proportion of population that benefits from the action | • 0: no individual  • 1: low proportion of affected individuals (<25% )  • 2 : moderate proportion (25-50% )  • 3: significant proportion (50-75%)  • 4 : majority of the population (> 75%) | | |
| **Social Impact Criteria (SIC)** | | | | | |
|  | SIC1 – Public acceptance | Level of public acceptance (agreement or non-agreement of the intervention by the population or stakeholders) | • -2: major disagreement  • -1: low disagreement  • 0: no effect  • 1: low agreement  • 2: important agreement | | |
|  | SIC2 – Impact to credibility | Impact to confidence in and credibility of organisation in charge (including adhesion to key messages) | • -3: significant increase in degree of confidence  • -2: moderate increase in degree of confidence  • -1: slight increase in degree of confidence  • 0: no effect  • 1: small reduction in degree of confidence  • 2: moderate reduction in degree of confidence  • 3: significant reduction in degree of confidence | | |
| **Economic Criteria (ECC)** | | | | | |
|  | ECC1 – Government cost | Cost to the government (national or province/state) | • 0: no cost  • 1: minimal costs (a few thousand)  • 2: moderate costs (hundreds of thousands)  • 3: high costs (millions) | | |
|  | ECC2 – Municipal cost | Cost to municipalities | • 0: no cost  • 1: minimal costs (a few thousand)  • 2: moderate costs (hundreds of thousands)  • 3: high costs (millions) | | |
|  | ECC3 – Individual cost | Cost to individuals and private sector | - 0 : no cost - 1 : minimal costs (individual <30$, private <100$) - 2 : moderate costs (individual 31-100$; private <100$) - 3 : high costs (individual >100$; private > 1000$) | | |
| **Strategic & Operational Criteria (SOC)** | | | | | |
|  | SOC1 - Delay | Delay before appearance of desired effect | • 0: no delay  • 1: very short term  • 2: short term  • 3 : medium  • 4: long term  • 5 : very long term | | |
|  | SOC2 – Complexity | Institutional and operational complexity of the action (including structural changes, hiring, etc.) | • 1: Simple (minor institutional changes)  • 2: Intermediate (requires hiring and further planning)  • 3: moderate (requires new working teams in a sector of intervention)  • 4: Complex (requires inter-sectoral / inter-institutional changes)  • 5: Very complex (requires the creation of new structures or organizations) | | |
|  | SOC3 – Sustainability | Sustainability of the action (or efficacy in time) | • 0: no duration  • 1: in days  • 2: in weeks  • 3: in months  • 4: in years | | |
|  | SOC4 – Other policy impact | Impact on other public policies (including potential conflicts with recommendations, economic efforts, etc.) | • -1: concordance / synergy  • 0: no conflict  • 1: low conflict  • 2: moderate conflicts  • 3: major conflicts | | |
| **Animal & Environmental Criteria (AEC)** | | | | | |
|  | AEC1 – Animal health impact | Impact on animal health and biodiversity  **= Type of effect * Scope * Value of species** | **Type of effect :**  • -1: health Improvement  • 0: no effect  • 1: morbidity  • 2 : mortality  **Scope (number of species affected) :**  • 1: no species  • 2: some species  • 3: several species | **Value of affected species (economic/ecological value, or endangered status) :**  • 1: low-value species or not at risk  • 2: species of low values ​​or susceptible species  • 3: moderate value or vulnerable/of concern species  • 4: important value of species or threatened/ endangered | |
|  | AEC2 – Environmental impact | Impact on physical environment and ecosystems  **= Type of effect * Scope * Value** | **Type of effect :**  • -1: improvement  • 0: no effect  • 1: low effect  • 2: moderate effects  • 3: High effects  **Geographic scope**:  • 1: none  • 2: small scale  • 3 large scale; | **Value** :  • 1: none  • 2 : terrestrial environment  • 3 : aquatic environment  • 4: terrestrial and aquatic environments  • 5: complex ecosystems (water – air - ground) | |
