# Supplementary material for: Assessing Interventions to Manage West Nile Virus Using Multi-Criteria Decision Analysis with Risk Scenarios
Source: PLoS One. 2016 Aug 5;11(8):e0160651. doi: 10.1371/journal.pone.0160651 (PMC4975439; doi:10.1371/journal.pone.0160651)
Supplement: S2 Table — (DOCX) [file pone.0160651.s006.docx]

**S2 Table. Matrix of evaluation scores for the interventions in the Quebec WNv management model**

PHC: Public health Criteria, SIC: Social impact criteria, ECC: economic criteria, SOC: strategic and operational criteria, AEC: animal and environmental health criteria.

| Criteria  Intervention | PHC-01 | PHC-02 | PHC-03 | PHC-04 | PHC-05 | PHC-06 | PHC-07 |  | SIC-01 | SIC-02 |  | ECC-01 | ECC-02 | ECC-03 |  | SOC-01 | SOC-02 | SOC-03 | SOC-04 |  | AEC-01 | AEC-02 |
| --- | --- | --- | --- | --- | --- | --- | --- | --- | --- | --- | --- | --- | --- | --- | --- | --- | --- | --- | --- | --- | --- | --- |
| INT-1 | 2 | 0 | 2 | 0 | 1 | 0 | 1 |  | -1 | 0 |  | 0 | 0 | 1 |  | 0 | 0 | 1 | 0 |  | 0 | 0 |
| INT-2 | 1 | 1 | 4 | 0 | 1 | 0 | 1 |  | 0 | 0 |  | 0 | 0 | 1 |  | 0 | 0 | 1 | 1 |  | 1 | 0 |
| INT-3 | 0 | 1 | 0 | 0 | 1 | 0 | 0 |  | 0 | 1 |  | 0 | 0 | 2 |  | 0 | 0 | 0 | 0 |  | 0 | 0 |
| INT-4 | 2 | 0 | -1 | 0 | 0 | 0 | 1 |  | 0 | 0 |  | 0 | 0 | 1 |  | 0 | 0 | 1 | 1 |  | 0 | 0 |
| INT-5 | 2 | 0 | 0 | 1 | 0 | 0 | 1 |  | -1 | 0 |  | 0 | 0 | 0 |  | 0 | 0 | 1 | 1 |  | 0 | 0 |
| INT-6 | 0 | 0 | -9 | -9 | 1 | 0 | 0 |  | 2 | 1 |  | 0 | 0 | 2 |  | 5 | 0 | 0 | 0 |  | 0 | 0 |
| INT-7 | 3 | 0 | 0 | 0 | 1 | 0 | 4 |  | 2 | 1 |  | 0 | 0 | 1 |  | 0 | 0 | 4 | 0 |  | 0 | 0 |
| INT-8 | 2 | 0 | 1 | 0 | 1 | 0 | 2 |  | -1 | 1 |  | 3 | 0 | 1 |  | 3 | 3 | 4 | 0 |  | 0 | 0 |
| INT-9 | 3 | 0 | 2 | 0 | 1 | 0 | 1 |  | -1 | 0 |  | 0 | 0 | 1 |  | 0 | 0 | 2 | 2 |  | 1 | 6 |
| INT-10 | 1 | 1 | 0 | 0 | 0 | 0 | 1 |  | 0 | 0 |  | 0 | 0 | 1 |  | 1 | 0 | 2 | 1 |  | 0 | 0 |
| INT-11 | 1 | 3 | 0 | 0 | 0 | 1 | 4 |  | 2 | -2 |  | 3 | 2 | 2 |  | 2 | 2 | 4 | 3 |  | 6 | 12 |
| INT-12 | 2 | 2 | 0 | 0 | -1 | 1 | 4 |  | 0 | 0 |  | 3 | 3 | 2 |  | 2 | 2 | 4 | 1 |  | 4 | 2 |
| INT-13 | 0 | 1 | 0 | 0 | 0 | 0 | 2 |  | -1 | 1 |  | 2 | 0 | 0 |  | 3 | 2 | 2 | 2 |  | 2 | 4 |
| INT-14 | 2 | 3 | 0 | 0 | 0 | 1 | 4 |  | 1 | 0 |  | 3 | 0 | 0 |  | 1 | 3 | 2 | 1 |  | 2 | 6 |
| INT-15 | 1 | 1 | 0 | 0 | 0 | 1 | 4 |  | -1 | 1 |  | 2 | 0 | 0 |  | 3 | 2 | 3 | 3 |  | 4 | 6 |
| INT-16 | 0 | 1 | 0 | 2 | 0 | 0 | 3 |  | -1 | 1 |  | 3 | 0 | 0 |  | 3 | 2 | 3 | 0 |  | 2 | 2 |
| INT-18 | 2 | 3 | 4 | 2 | 0 | 1 | 4 |  | -2 | 2 |  | 3 | 0 | 0 |  | 1 | 4 | 1 | 3 |  | 12 | 8 |
| INT-19 | 0 | 1 | 0 | 0 | 0 | 4 | 2 |  | 0 | 0 |  | 2 | 0 | 0 |  | 1 | 2 | 4 | 0 |  | 0 | 0 |
| INT-23 | -1 | -1 | 0 | 0 | 1 | -1 | 0 |  | 0 | -2 |  | 0 | 0 | 0 |  | 5 | 0 | 0 | 0 |  | 0 | 0 |
