# Supplementary material for: Assessing Interventions to Manage West Nile Virus Using Multi-Criteria Decision Analysis with Risk Scenarios
Source: PLoS One. 2016 Aug 5;11(8):e0160651. doi: 10.1371/journal.pone.0160651 (PMC4975439; doi:10.1371/journal.pone.0160651)
Supplement: S3 Table — (DOCX) [file pone.0160651.s007.docx]

**S3 Table. Stakeholder weighting results by criteria and category for the Scenarios 1& 2 (low risk transmission)**

PHC: Public health Criteria, SIC: Social impact criteria, ECC: economic criteria, SOC: strategic and operational criteria, AEC: animal and environmental health criteria. S1-S12 – stakeholders 1-12.

| **Scenario 1** | **S1** | | **S2** |  | **S3** |  | **S4** |  | **S5** |  | **S6** |  | **S7** |  | **S8** |  | **S9** |  | **S10** |  | **S11** |  | **S12** | |  |
| --- | --- | --- | --- | --- | --- | --- | --- | --- | --- | --- | --- | --- | --- | --- | --- | --- | --- | --- | --- | --- | --- | --- | --- | --- | --- |
| PHC-01 | 16 | 40 | 10 | 50 | 9 | 30 | 5 | 50 | 11 | 50 | 15 | 50 | 16 | 40 | 6.25 | 25 | 6.67 | 66.69 | 16 | 40 | 16.8 | 48 | 0 | 55 |  |
| PHC-02 | 8 |  | 10 |  | 3 |  | 5 |  | 11 |  | 0 |  | 8 |  | 5 |  | 6.67 |  | 6 |  | 4.8 |  | 0 |  |  |
| PHC-03 | 4 |  | 5 |  | 3 |  | 5 |  | 3 |  | 20 |  | 6 |  | 7.5 |  | 6.67 |  | 10 |  | 2.4 |  | 25 |  |  |
| PHC-04 | 4 |  | 5 |  | 3 |  | 5 |  | 3 |  | 10 |  | 2 |  | 1.25 |  | 6.67 |  | 2 |  | 0.96 |  | 20 |  |  |
| PHC-05 | 2 |  | 5 |  | 3 |  | 5 |  | 1 |  | 2.5 |  | 4 |  | 1.25 |  | 6.67 |  | 2 |  | 2.4 |  | 5 |  |  |
| PHC-06 | 2 |  | 5 |  | 3 |  | 5 |  | 1 |  | 0 |  | 0 |  | 1.875 |  | 6.67 |  | 2 |  | 4.8 |  | 0 |  |  |
| PHC-07 | 4 |  | 10 |  | 6 |  | 20 |  | 20 |  | 2.5 |  | 4 |  | 1.875 |  | 26.67 |  | 2 |  | 15.84 |  | 5 |  |  |
| SIC-01 | 2 | 5 | 5 | 10 | 7 | 10 | 5 | 10 | 2.5 | 5 | 5 | 10 | 10 | 10 | 6.4 | 8 | 0 | 0 | 5 | 10 | 3 | 10 | 5 | 10 |  |
| SIC-02 | 3 |  | 5 |  | 3 |  | 5 |  | 2.5 |  | 5 |  | 0 |  | 1.6 |  | 0 |  | 5 |  | 7 |  | 5 |  |  |
| ECC-01 | 7.5 | 25 | 7.5 | 15 | 5 | 20 | 6.25 | 25 | 6.8 | 20 | 6.25 | 25 | 7.5 | 15 | 44 | 55 | 8.33 | 33.33 | 10 | 25 | 8 | 20 | 25 | 25 |  |
| ECC-02 | 2.5 |  | 5.25 |  | 5 |  | 6.25 |  | 6.6 |  | 6.25 |  | 3.75 |  | 8.25 |  | 8.33 |  | 7.5 |  | 7 |  | 0 |  |  |
| ECC-03 | 15 |  | 2.25 |  | 10 |  | 12.5 |  | 6.6 |  | 12.5 |  | 3.75 |  | 2.75 |  | 16.67 |  | 7.5 |  | 5 |  | 0 |  |  |
| SOC-01 | 6 | 20 | 4 | 20 | 5.25 | 15 | 0 | 15 | 7.5 | 15 | 2.5 | 10 | 5 | 25 | 3 | 10 | 0 | 0 | 0.5 | 5 | 5 | 20 | 2 | 8 |  |
| SOC-02 | 4 |  | 7 |  | 2.25 |  | 7.5 |  | 3.75 |  | 2.5 |  | 7.5 |  | 3 |  | 0 |  | 1 |  | 4 |  | 2 |  |  |
| SOC-03 | 8 |  | 7 |  | 6 |  | 7.5 |  | 1.95 |  | 2.5 |  | 5 |  | 2.5 |  | 0 |  | 3 |  | 5 |  | 2 |  |  |
| SOC-04 | 2 |  | 2 |  | 1.5 |  | 0 |  | 1.8 |  | 2.5 |  | 7.5 |  | 1.5 |  | 0 |  | 0.5 |  | 6 |  | 2 |  |  |
| AEC-01 | 5 | 10 | 2.5 | 5 | 10 | 25 | 0 | 0 | 5 | 10 | 2.5 | 5 | 5 | 10 | 0.8 | 2 | 0 | 0 | 10 | 20 | 1.2 | 2 | 1 | 2 |  |
| AEC-02 | 5 |  | 2.5 |  | 15 |  | 0 |  | 5 |  | 2.5 |  | 5 |  | 1.2 |  | 0 |  | 10 |  | 0.8 |  | 1 |  |  |
| **Scenario 2** | **S1** | | **S2** | | **S3** | | **S4** | | **S5** | | **S6** | | **S7** | | **S8** | | **S9** | | **S10** | | **S11** | | **S12** | |  |
| PHC-01 | 16 | 40 | 10 | 50 | 9 | 30 | 12 | 30 | 11 | 50 | 15 | 50 | 16 | 40 | 14 | 40 | 0 | 10 | 8.75 | 25 | 16.96 | 53 | 0 | 55 |  |
| PHC-02 | 8 |  | 10 |  | 3 |  | 12 |  | 11 |  | 0 |  | 8 |  | 6 |  | 0 |  | 5 |  | 5.3 |  | 0 |  |  |
| PHC-03 | 4 |  | 5 |  | 3 |  | 0 |  | 3 |  | 20 |  | 4 |  | 4 |  | 8 |  | 7.5 |  | 2.65 |  | 25 |  |  |
| PHC-04 | 4 |  | 5 |  | 3 |  | 6 |  | 3 |  | 10 |  | 4 |  | 2 |  | 2 |  | 2 |  | 2.65 |  | 20 |  |  |
| PHC-05 | 2 |  | 5 |  | 3 |  | 0 |  | 1 |  | 2.5 |  | 4 |  | 2 |  | 0 |  | 0.5 |  | 2.65 |  | 5 |  |  |
| PHC-06 | 2 |  | 5 |  | 3 |  | 0 |  | 1 |  | 0 |  | 0 |  | 6 |  | 0 |  | 0.5 |  | 2.65 |  | 0 |  |  |
| PHC-07 | 4 |  | 10 |  | 6 |  | 0 |  | 20 |  | 2.5 |  | 4 |  | 6 |  | 0 |  | 0.75 |  | 20.14 |  | 5 |  |  |
| SIC-01 | 2 | 5 | 5 | 10 | 7 | 10 | 10 | 20 | 2.5 | 5 | 5 | 10 | 5 | 10 | 8 | 10 | 0 | 0 | 2 | 10 | 5.25 | 15 | 5 | 10 |  |
| SIC-02 | 3 |  | 5 |  | 3 |  | 10 |  | 2.5 |  | 5 |  | 5 |  | 2 |  | 0 |  | 8 |  | 9.75 |  | 5 |  |  |
| ECC-01 | 7.5 | 25 | 7.5 | 15 | 5 | 20 | 0 | 10 | 6.8 | 20 | 6.25 | 25 | 10 | 20 | 32 | 40 | 40 | 80 | 10 | 25 | 1.75 | 5 | 25 | 25 |  |
| ECC-02 | 2.5 |  | 5.25 |  | 5 |  | 5 |  | 6.6 |  | 6.25 |  | 5 |  | 6 |  | 40 |  | 7.5 |  | 1.75 |  | 0 |  |  |
| ECC-03 | 15 |  | 2.25 |  | 10 |  | 5 |  | 6.6 |  | 12.5 |  | 5 |  | 2 |  | 0 |  | 7.5 |  | 1.5 |  | 0 |  |  |
| SOC-01 | 6 | 20 | 4 | 20 | 5.25 | 15 | 9 | 30 | 7.5 | 15 | 2.5 | 10 | 5 | 20 | 2.4 | 8 | 0 | 0 | 6 | 20 | 6.25 | 25 | 2 | 8 |  |
| SOC-02 | 4 |  | 7 |  | 2.25 |  | 9 |  | 3.75 |  | 2.5 |  | 5 |  | 2.4 |  | 0 |  | 6 |  | 7.5 |  | 2 |  |  |
| SOC-03 | 8 |  | 7 |  | 6 |  | 12 |  | 1.95 |  | 2.5 |  | 5 |  | 2 |  | 0 |  | 6 |  | 5 |  | 2 |  |  |
| SOC-04 | 2 |  | 2 |  | 1.5 |  | 0 |  | 1.8 |  | 2.5 |  | 5 |  | 1.2 |  | 0 |  | 2 |  | 6.25 |  | 2 |  |  |
| AEC-01 | 5 | 10 | 2.5 | 5 | 10 | 25 | 3 | 10 | 5 | 10 | 2.5 | 5 | 5 | 10 | 0.8 | 2 | 1 | 10 | 10 | 20 | 1.2 | 2 | 1 | 2 |  |
| AEC-02 | 5 |  | 2.5 |  | 15 |  | 7 |  | 5 |  | 2.5 |  | 5 |  | 1.2 |  | 9 |  | 10 |  | 0.8 |  | 1 |  |  |
