# Supplementary material for: Assessing Interventions to Manage West Nile Virus Using Multi-Criteria Decision Analysis with Risk Scenarios
Source: PLoS One. 2016 Aug 5;11(8):e0160651. doi: 10.1371/journal.pone.0160651 (PMC4975439; doi:10.1371/journal.pone.0160651)
Supplement: S5 Table — (DOCX) [file pone.0160651.s009.docx]

**S5 Table. Stakeholder weighting results by criteria and category for the Scenarios 5& 6 (high risk transmission)**

PHC: Public health Criteria, SIC: Social impact criteria, ECC: economic criteria, SOC: strategic and operational criteria, AEC: animal and environmental health criteria. S1-S12 – stakeholders 1-12.

| **Scenario 5** | **S1** | | **S2** | | **S3** | | **S4** | | **S5** | | **S6** | | **S7** | | **S8** | | **S9** | | **S10** | | **S11** | | **S12** | |
| --- | --- | --- | --- | --- | --- | --- | --- | --- | --- | --- | --- | --- | --- | --- | --- | --- | --- | --- | --- | --- | --- | --- | --- | --- |
| PHC-01 | 16 | 40 | 10 | 50 | 15 | 50 | 8 | 40 | 11 | 50 | 63 | 90 | 14 | 40 | 18 | 60 | 13 | 65 | 6 | 30 | 17.6 | 55 | 40 | 60 |
| PHC-02 | 8 |  | 10 |  | 7.5 |  | 0 |  | 11 |  | 0 |  | 8 |  | 15 |  | 0 |  | 4.5 |  | 11 |  | 0 |  |
| PHC-03 | 4 |  | 5 |  | 5 |  | 8 |  | 3 |  | 27 |  | 8 |  | 21 |  | 35.75 |  | 9 |  | 2.75 |  | 20 |  |
| PHC-04 | 4 |  | 5 |  | 5 |  | 8 |  | 3 |  | 0 |  | 4 |  | 0 |  | 16.25 |  | 3 |  | 1.1 |  | 0 |  |
| PHC-05 | 2 |  | 5 |  | 5 |  | 0 |  | 1 |  | 0 |  | 2 |  | 0 |  | 0 |  | 1.5 |  | 1.65 |  | 0 |  |
| PHC-06 | 2 |  | 5 |  | 7.5 |  | 8 |  | 1 |  | 0 |  | 2 |  | 3 |  | 0 |  | 3 |  | 5.5 |  | 0 |  |
| PHC-07 | 4 |  | 10 |  | 5 |  | 8 |  | 20 |  | 0 |  | 2 |  | 3 |  | 0 |  | 3 |  | 15.4 |  | 0 |  |
| SIC-01 | 2 | 5 | 5 | 10 | 3.5 | 5 | 15 | 30 | 2.5 | 5 | 2.5 | 5 | 10 | 20 | 1.4 | 2 | 12.5 | 25 | 12.5 | 25 | 1.75 | 5 | 0 | 20 |
| SIC-02 | 3 |  | 5 |  | 1.5 |  | 15 |  | 2.5 |  | 2.5 |  | 10 |  | 0.6 |  | 12.5 |  | 12.5 |  | 3.25 |  | 20 |  |
| ECC-01 | 7.5 | 25 | 5 | 10 | 2.5 | 10 | 4 | 10 | 6.8 | 20 | 5 | 5 | 10 | 20 | 27 | 30 | 0 | 0 | 10 | 20 | 8 | 20 | 5 | 5 |
| ECC-02 | 2.5 |  | 3.5 |  | 2.5 |  | 4 |  | 6.6 |  | 0 |  | 5 |  | 3 |  | 0 |  | 6 |  | 7 |  | 0 |  |
| ECC-03 | 15 |  | 1.5 |  | 5 |  | 2 |  | 6.6 |  | 0 |  | 5 |  | 0 |  | 0 |  | 4 |  | 5 |  | 0 |  |
| SOC-01 | 6 | 20 | 5 | 25 | 8.75 | 25 | 0 | 10 | 7.5 | 15 | 0 | 0 | 1.5 | 15 | 1.5 | 6 | 0 | 0 | 1.5 | 5 | 6 | 15 | 15 | 15 |
| SOC-02 | 4 |  | 8.75 |  | 3.75 |  | 5 |  | 3.75 |  | 0 |  | 6 |  | 3.6 |  | 0 |  | 1.25 |  | 2.25 |  | 0 |  |
| SOC-03 | 8 |  | 8.75 |  | 10 |  | 5 |  | 1.95 |  | 0 |  | 6 |  | 0.9 |  | 0 |  | 1.75 |  | 2.25 |  | 0 |  |
| SOC-04 | 2 |  | 2.5 |  | 2.5 |  | 0 |  | 1.8 |  | 0 |  | 1.5 |  | 0 |  | 0 |  | 0.5 |  | 4.5 |  | 0 |  |
| AEC-01 | 5 | 10 | 2.5 | 5 | 4 | 10 | 5 | 10 | 5 | 10 | 0 | 0 | 2.5 | 5 | 0.8 | 2 | 3 | 10 | 8 | 20 | 2.5 | 5 | 0 | 0 |
| AEC-02 | 5 |  | 2.5 |  | 6 |  | 5 |  | 5 |  | 0 |  | 2.5 |  | 1.2 |  | 7 |  | 12 |  | 2.5 |  | 0 |  |
| **Scenario 6** | **S1** | | **S2** | | **S3** | | **S4** | | **S5** | | **S6** | | **S7** | | **S8** | | **S9** | | **S10** | | **S11** | | **S12** | |
| PHC-01 | 16 | 40 | 10 | 50 | 15 | 50 | 8 | 40 | 11 | 50 | 16 | 40 | 14 | 40 | 24 | 80 | 13 | 65 | 5 | 25 | 15.4 | 55 | 40 | 60 |
| PHC-02 | 8 |  | 10 |  | 7.5 |  | 0 |  | 11 |  | 0 |  | 8 |  | 24 |  | 0 |  | 3.75 |  | 11 |  | 0 |  |
| PHC-03 | 4 |  | 5 |  | 5 |  | 8 |  | 3 |  | 8 |  | 8 |  | 28 |  | 35.75 |  | 7.5 |  | 2.75 |  | 20 |  |
| PHC-04 | 4 |  | 5 |  | 5 |  | 8 |  | 3 |  | 8 |  | 4 |  | 0 |  | 16.25 |  | 1.25 |  | 1.1 |  | 0 |  |
| PHC-05 | 2 |  | 5 |  | 5 |  | 0 |  | 1 |  | 4 |  | 2 |  | 0 |  | 0 |  | 2.5 |  | 1.65 |  | 0 |  |
| PHC-06 | 2 |  | 5 |  | 7.5 |  | 8 |  | 1 |  | 0 |  | 2 |  | 2.4 |  | 0 |  | 2.5 |  | 5.5 |  | 0 |  |
| PHC-07 | 4 |  | 10 |  | 5 |  | 8 |  | 20 |  | 4 |  | 2 |  | 1.6 |  | 0 |  | 2.5 |  | 17.6 |  | 0 |  |
| SIC-01 | 2 | 5 | 5 | 10 | 3.5 | 5 | 10 | 20 | 2.5 | 5 | 5 | 10 | 10 | 20 | 0.7 | 1 | 12.5 | 25 | 8 | 20 | 5.25 | 15 | 0 | 20 |
| SIC-02 | 3 |  | 5 |  | 1.5 |  | 10 |  | 2.5 |  | 5 |  | 10 |  | 0.3 |  | 12.5 |  | 12 |  | 9.75 |  | 20 |  |
| ECC-01 | 7.5 | 25 | 5 | 10 | 2.5 | 10 | 8 | 20 | 6.8 | 20 | 30 | 30 | 7.5 | 15 | 13.5 | 15 | 0 | 0 | 15 | 25 | 2 | 5 | 5 | 5 |
| ECC-02 | 2.5 |  | 3.5 |  | 2.5 |  | 8 |  | 6.6 |  | 0 |  | 3.75 |  | 1.5 |  | 0 |  | 7.5 |  | 1.75 |  | 0 |  |
| ECC-03 | 15 |  | 1.5 |  | 5 |  | 4 |  | 6.6 |  | 0 |  | 3.75 |  | 0 |  | 0 |  | 2.5 |  | 1.25 |  | 0 |  |
| SOC-01 | 6 | 20 | 5 | 25 | 8.75 | 25 | 0 | 10 | 7.5 | 15 | 2.5 | 10 | 1.5 | 15 | 0.6 | 3 | 0 | 0 | 6 | 20 | 8 | 20 | 15 | 15 |
| SOC-02 | 4 |  | 8.75 |  | 3.75 |  | 5 |  | 3.75 |  | 2.5 |  | 4.5 |  | 1.8 |  | 0 |  | 5 |  | 3 |  | 0 |  |
| SOC-03 | 8 |  | 8.75 |  | 10 |  | 5 |  | 1.95 |  | 2.5 |  | 7.5 |  | 0.6 |  | 0 |  | 7 |  | 3 |  | 0 |  |
| SOC-04 | 2 |  | 2.5 |  | 2.5 |  | 0 |  | 1.8 |  | 2.5 |  | 1.5 |  | 0 |  | 0 |  | 2 |  | 6 |  | 0 |  |
| AEC-01 | 5 | 10 | 2.5 | 5 | 4 | 10 | 5 | 10 | 5 | 10 | 5 | 10 | 5 | 10 | 0.4 | 1 | 3 | 10 | 5 | 10 | 2.5 | 5 | 0 | 0 |
| AEC-02 | 5 |  | 2.5 |  | 6 |  | 5 |  | 5 |  | 5 |  | 5 |  | 0.6 |  | 7 |  | 5 |  | 2.5 |  | 0 |  |
